# A quantitative model of the initiation of DNA replication in *Saccharomyces cerevisiae* predicts the effects of system perturbations

**DOI:** 10.1186/1752-0509-6-78

**Published:** 2012-06-27

**Authors:** Rohan D Gidvani, Peter Sudmant, Grace Li, Lance F DaSilva, Brendan J McConkey, Bernard P Duncker, Brian P Ingalls

**Affiliations:** 1 Department of Biology, University of Waterloo, Waterloo, ON, Canada; 2 Department of Applied Mathematics, University of Waterloo, Waterloo, ON, Canada

## Abstract

**Background:**

Eukaryotic cell proliferation involves DNA replication, a tightly regulated process mediated by a multitude of protein factors. In budding yeast, the initiation of replication is facilitated by the heterohexameric origin recognition complex (ORC). ORC binds to specific origins of replication and then serves as a scaffold for the recruitment of other factors such as Cdt1, Cdc6, the Mcm2-7 complex, Cdc45 and the Dbf4-Cdc7 kinase complex. While many of the mechanisms controlling these associations are well documented, mathematical models are needed to explore the network’s dynamic behaviour. We have developed an ordinary differential equation-based model of the protein-protein interaction network describing replication initiation.

**Results:**

The model was validated against quantified levels of protein factors over a range of cell cycle timepoints. Using chromatin extracts from synchronized *Saccharomyces cerevisiae* cell cultures, we were able to monitor the *in vivo* fluctuations of several of the aforementioned proteins, with additional data obtained from the literature. The model behaviour conforms to perturbation trials previously reported in the literature, and accurately predicts the results of our own knockdown experiments. Furthermore, we successfully incorporated our replication initiation model into an established model of the entire yeast cell cycle, thus providing a comprehensive description of these processes.

**Conclusions:**

This study establishes a robust model of the processes driving DNA replication initiation. The model was validated against observed cell concentrations of the driving factors, and characterizes the interactions between factors implicated in eukaryotic DNA replication. Finally, this model can serve as a guide in efforts to generate a comprehensive model of the mammalian cell cycle in order to explore cancer-related phenotypes.

## Background

The machinery of the eukaryotic cell cycle has been extensively dissected and described, in both simple and complex organisms. Proliferation hinges on the cell’s ability to replicate the genome with high fidelity, segregate the chromosomes equally, and ultimately divide into two genetically identical cells. A fundamental process in the regulation of DNA replication is the step-wise assembly of the pre-replicative complex (pre-RC) at origins of replication. The pre-RC is a congregation of proteins each performing a specific role. Its formation is facilitated by the six-subunit origin recognition complex (ORC), which, in the budding yeast *Saccharomyces cerevisiae,* binds an 11 bp consensus sequence [1-3]. ORC then recruits Cdc6, which, like ORC, exhibits ATPase activity [4-6]. The co-import of Cdt1 and the Mcm2-7 complex (MCM) into the nucleus follows [7], and the MCM·Cdt1 heptamer is then targeted to origins by an interaction between Cdt1 and Orc6 [8,9]. Initial loading of an MCM ring at the origin requires Cdc6 ATP-hydrolysis. Reiterative loading of an additional MCM molecule occurs via ORC ATP-hydrolysis [10], resulting in two rings at each origin [11-13]. At this point origins are said to be licensed. In late G1 phase, a burst of Dbf4 synthesis activates the Dbf4-dependent kinase Cdc7 (DDK), which then phosphorylates multiple MCM subunits [14-18], bringing about a conformational change that stimulates MCM helicase activity. Dbf4 levels decrease over the course of S-phase and, starting at the metaphase/anaphase transition, Dbf4 is actively degraded by the anaphase promoting complex (APC) and its activating co-factor, Cdc20 [19-23]. In this way, Dbf4 levels are prevented from rising until the next G1/S transition.

The phosphorylation of MCM by DDK is coincident with the phosphorylation of the protein factors Sld2 and Sld3 by Clb5-Cdc28, a cyclin-dependent kinase (CDK) complex, the activity of which rises just prior to S-phase entry. The Sld proteins, once phosphorylated, are stabilized as a complex with the adaptor protein Dpb11 and the tetrameric GINS complex, forming a module that interacts with Cdc45. The latter acts as a scaffold for this module, which is then competent to associate with the pre-RC and attract DNA polymerase [15,24-26]. A recent study shows that the end result is the tight association of Cdc45, MCM and GINS (collectively known as CMG) with origins, allowing the unwinding of DNA and processive replication by DNA polymerase [27]. This represents the essential role of CDK in stabilizing polymerase at the moving replication fork and switching the system from a pre-replicative state to a replicative one. From this point until late in mitosis, CDK levels remain high. This continued CDK activity prevents re-establishment of pre-RCs at origins that have already fired through a number of mechanisms. Firstly, CDK phosphorylates Cdc6, thus causing the SCF^cdc4^ complex to target Cdc6 to the proteasome for degradation [28-31]. Secondly, Orc2 and Orc6 are phosphorylated by CDK [32-34], with the phosphorylation of Orc6 rendering it refractory to interaction with Cdt1 [35], thereby preventing further MCM loading. Finally, CDK facilitates the nuclear export of both MCM and Cdt1, at different time points. Just prior to initiation, Cdt1 exits via a CDK-dependent mechanism, while MCM proteins fall off the DNA upon fork termination and are then exported in a CDK-dependent manner [7,36-38]. Thus, while CDK initiates replication, it subsequently prevents pre-RC reassembly. This illustrates its dual role in triggering initiation through formation of CMG, then preventing re-initiation by inhibiting pre-RC reformation.

Mathematical modeling has been successfully used in the past to address various aspects of the cell cycle. Early models (e.g. [39]) did not incorporate specific biochemical mechanisms; they were hypothetical representations of periodic cellular activity. As the molecular mechanisms driving the cell cycle were revealed, models appeared that incorporated these findings (e.g. [40-43]). For *S. cerevisiae* in particular, multiple modeling approaches have been applied, based both on network descriptions [44] and on specific molecular details such as gene expression and biochemical kinetics [45-47] (reviewed in [48]). Some modeling efforts have been comprehensive, such as the Tyson group’s ordinary differential equation (ODE)-based models [45,46], while others address specific cell-cycle phenomena, such as the links between cell size and cycle progression [49,50]. Spiesser et al. [51] developed a model of chromosomal replication, which reproduced the spatio-temporal replication profile of yeast chromosomes. Origin firing was also described in [52,53] wherein the authors used a stochastic model to describe these origin-specific features of replication.

A recent report [54] presented an ODE-based model describing the initiation of DNA replication, incorporating origin licensing, firing and the network of regulatory phosphorylation events. The model parameters were partly calibrated against experimental data, but largely selected through an optimization routine designed to attain an idealized function, resulting in a model that is particularly suited to exploring events specifically at the G1/S transition.

Here, we present a new model of the initiation of DNA replication. In contrast to the work of Brümmer et al. [54], we took a ‘bottom-up’ approach and began by gathering *in vivo* data for precise protein levels at specific cell cycle time-points, then calibrated our model against these values. Rather than limiting ourselves to the observation of firing near the G1/S transition and fitting to DNA-specific replication profiles, we validated our model against the behaviour of the constituent protein complexes throughout the entire cell cycle. To facilitate the use of our model in a comprehensive description of the cell cycle, we designed it to integrate easily with the model of Chen et al. [45]. Finally, we validated the model by comparing *in silico* predictions to experimental observations, using both our own knockdown experiments and results from the literature. The model presented here consolidates the known interactions between DNA replication initiation proteins and the mechanisms that allow them to drive genome duplication. Additionally, regulatory aspects of the system, which ensure that re-replication does not occur, have been modeled. The model’s behaviour provides a falsifiable hypothesis regarding the dynamics of DNA replication initiation. Furthermore, it accurately predicts the phenotypes of known experimental cell cycle mutants as well as those arising through *in vivo* perturbations to proteins in the network. Because our model is constrained only by the fluctuating levels of replication factors, it provides a unique understanding of the kinetics governing the reactions between them. Successful integration into a whole cell cycle model allows the initiation of DNA replication to be explored in a broader quantitative context.

## Results and discussion

**Figure 1:**
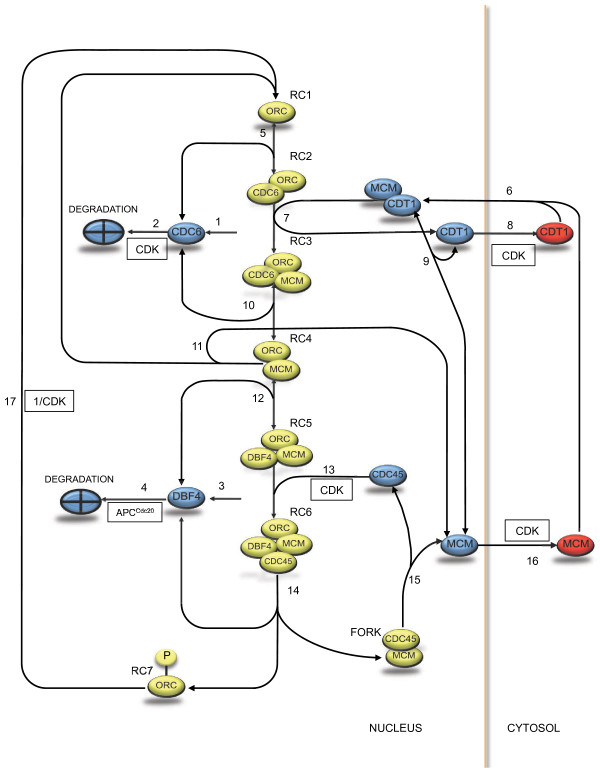
**Network diagram for the initiation of DNA replication.** Chromatin-bound species are shown in yellow. Reactions that we have considered reversible are shown with an arrowhead at each end. ORC-bound DNA (RC1) specifies a complex that has bound origin sequences following DNA replication of the previous cycle. Cdc6 reversibly binds ORC-bound DNA starting in late M-phase to form RC2. The Mcm2-7 hexamers, chaperoned by Cdt1 are localized to origins where they are loaded onto the double helix (RC3). Cdt1 is later exported from the nucleus by a CDK-dependent mechanism (i.e. by Clb5-Cdc28). Free Cdc6 is targeted for proteolysis in a CDK-dependent manner. Upon Cdc6 dissociation, the complex of MCM and ORC (RC4) is also subject to dissociation. RC4 awaits association of and activation by a complex of Dbf4 and Cdc7 (DDK), which phosphorylates various MCM subunits (RC5). Required ultimately for the stabilization of DNA polymerase, Cdc45 binds in response to specific CDK phosphorylation events (RC6, also called the Pre-IC). DNA replication begins as forks are established (FORK). Dbf4 dissociates soon after initiation and is constitutively degraded throughout S-phase. Its levels cannot rise until late G1 since it is actively targeted for degradation by APC^Cdc20^, whose low in G1 are sufficient for this inhibition. Once a replication fork terminates, both Cdc45 and the MCM fall off the chromatin. Free MCM is exported to the cytoplasm via a CDK-dependent mechanism. ORC is phosphorylated by CDK (RC7) and cannot interact with pre-RC components until it is dephosphorylated, returning it to the RC1 state.

We began construction of our model by identifying the important players in replication initiation and establishing an interaction network, as shown in Figure 1. After selecting appropriate descriptions of reaction kinetics, we generated an ODE-based model and calibrated the model parameters to *in vivo* data.

### Description of model components

The model describes sixteen molecular species (twelve of which are dynamically independent) and depends on twenty-four parameters, which characterize the rates of seventeen biochemical processes (protein expression and degradation, complex association/dissociation, and transport across the nuclear membrane). The model describes the following molecular species (Figure 1).

RC1 (Replication complex, state 1): origin-bound ORC

RC2: origin-bound ORC associated with CDC6

RC3: origin-bound ORC associated with CDC6, with MCM loaded

RC4: origin-bound ORC, with MCM loaded

RC5: origin-bound ORC, with MCM loaded and DBF4 associated

RC6: origin-bound ORC, with MCM loaded and DBF4 and CDC45 associated

RC7: origin-bound phosphorylated ORC

FORK: the elongation fork, with MCM and CDC45 associated

CDC6N: non-chromatin associated nuclear Cdc6

DBF4N: non-chromatin associated nuclear Dbf4

CDC45N: non-chromatin associated nuclear Cdc45

MCMC: cytosolic MCM

CDT1C: cytosolic CDT1

MCM·CDT1N: non-chromatin associated nuclear MCM bound to Cdt1

CDT1N: non-chromatin associated nuclear Cdt1

MCMN: non-chromatin associated nuclear MCM

The MCM species corresponds to dimers of Mcm2-7 heterohexamers, as two complexes are loaded at each origin. Similarly, the CDC45 species corresponds to a dimer, as described by Bowers et al. [11]. We describe concentrations in units of molecules per cell.

### Reaction events

The seventeen processes that make up the model are shown in Table 1. Their rates depend on the species concentrations, the model parameters, and on two fixed, time-varying input functions describing the abundance of Clb5 (representing activated CDK) and of Cdc20.

**Table 1:** Kinetic reaction rates describing the network

**Rate**	**Description**	**Rate equation**
	*Expression & Degradation*	
v_1_	Expression of CDC6	k_1_
v_2_	Degradation of CDC6	k_2_CLB5·CDC6
v_3_	Expression of DBF4	k_3_
v_4_	Degradation of DBF4	k_4_DBF4·CDC20
	*Formation of the Pre-Replicative Complex*	
v_5_	Association of ORC and CDC6	k_5_RC1·CDC6 – k_5r_RC2
v_6_	Association and nuclear import of MCM and CDT1	k_6_MCM_C_·CDT1_C_/(KM_1_ + MCM_C_)
v_7_	Loading of MCM by CDT1	k_7_RC2·MCM·CDT1
v_8_	Nuclear export of CDT1	k_8_CLB5·CDT1
v_9_	Dissociation of nuclear MCM-CDT1 complex	k_9_MCM·CDT1 – k_9r_MCM·CDT1
v_10_	Dissociation of CDC6 from the Pre-RC	k_10_RC3 – k_10r_CDC6·RC4
	*Formation of the Pre-Initiation Complex*	
v_11_	Dissociation of ORC and MCM from Pre-RC	k_11_RC4
v_12_	Association of DBF4 and the Pre-RC	k_12_RC4·DBF4 – k_12r_RC5
v_13_	Association of CDC45 and the Pre-RC	k_13_RC5·CDC45·CLB5/(KM_2_ + CDC45)
	*Post-Replicative Complex and Ensuing Events*	
v_14_	Origin firing	k_14_RC6
v_15_	Breakup of the elongation fork	k_15_FORK
v_16_	Nuclear export of MCM	k_16_MCM·CLB5
v_17_	Phosphorylation of ORC	k_17_RC7/(1 + (CLB5/k_18_)^5^)

In choosing reaction kinetics, we balanced the complexity of the model against its ability to adequately describe the behaviour of the overall system. We limited our description of initiation to the interactions between the pre-RC and replisome proteins that we found to be the essential core of the network (e.g. Dbf4 representing the Dbf4-Cdc7 complex, discussed below). As a result, certain processes were combined into single events, some reactions were presumed irreversible, and only some reaction rates were presumed to have non-linear kinetics.

Except for RC7, phosphorylation states are not explicitly described, as we have no data for the individual phosphorylation events. This is acceptable for our purposes as the lumped function of CDK in each case is consistent with a scenario where the effect of CDK is proportional to its concentration (i.e. [Clb5]). Additionally, processes that involve multi-protein complexes are represented by a single member – one CMG (Cdc45·Mcm2-7·GINS) complex stabilizes DNA polymerase at each replication fork. Of the three protein factors it is comprised of, Cdc45 is limiting. Although MCM is also included in the GINS complex, we model both MCM and Cdc45 as separate species. Dbf4 represents the Dbf4-Cdc7 kinase complex and Mcm2 represents the Mcm2-7 helicase. Although the protein factors Cdc45, Dbp11, Sld2, Sld3 and GINS interact to facilitate formation of the pre-initiation complex at origins, we model only Cdc45, which is the limiting factor in the CMG complex [55]; ultimately the number of forks fired (described by our model) is dependent on the Cdc45 concentration. We take Dbf4, which is the limiting regulatory subunit of Cdc7, as representative of active DDK, which is one of the limiting factors in replication initiation [56]. Mcm2 is used to represent MCM complexes; the Mcm2 concentration has been reported to approximate the number of total complexes per cell in an asynchronous population [57,58]. The replication complexes in our models exist only on chromatin and therefore represent the activity of these proteins at the DNA as opposed to soluble complexes.

The network shown in Figure 1 includes both reversible and irreversible reactions as indicated. Association/dissociation reactions are considered reversible, in accordance with a dynamic pre-RC/pre-IC loading mechanism as described above. In most cases, phosphorylation events are modeled as irreversible, in the absence of identified countervailing enzymes. We found that it was sufficient to describe most reaction rates by mass action kinetics. In cases where saturation occurs (the nuclear import of MCM·Cdt1, v_6_, and the association of Cdc45 with ORC, v_13_), we employed Michaelis-Menten kinetics. To simplify the description of the phosphorylation of ORC by CDK (RC7), we do not describe phosporylation and dephosphorylation explicitly, but combine them into a single dephosphorylation event whose rate is inversely proportional to the level of CDK (v_17_). We introduce cooperativity in this mechanism to account for multiple phosphorylation events [32] or an additional inhibitory CDK-Orc6 binding mechanism [34,35].

The establishment of replication complexes in our model reflects the sequential binding of proteins that constitute the pre-replicative complex. In some cases the association and dissociation of pre-RC components is reversible. We treat the loading and maintenance of Mcm2-7 helicase complexes as a dynamic process, which is dependent on the concentrations of the factors ORC, Cdc6, Cdt1 and Mcm2-7 itself. A mechanistic model for the dynamic assembly of pre-RCs was first described by the Bell lab [59]. The requirement of pre-RC factors for maintenance of helicase-loaded origins in late G1 has been further demonstrated by work from these researchers as well as our group [8,9,60].

### Network and differential equations

Referring to Figure 1 and Table 1, the dynamics of the system are described as:

(1) $$\begin{array}{ll} \frac{dRC2}{dt}=v_{5}-v_{7} & \frac{dFORK}{dt}=v_{14}-v_{15}\\ \frac{dRC3}{dt}=v_{7}-v_{10} & \frac{dCDC6_{N}}{dt}=v_{1}+v_{10}-v_{5}-v_{2}\\ \frac{dRC4}{dt}=v_{10}-v_{12}-v_{11} & \frac{dDBF4_{N}}{dt}=v_{3}+v_{14}-v_{12}-v_{4}\\ \frac{dRC5}{dt}=v_{12}-v_{13} & \frac{dCDT1_{N}}{dt}=v_{7}-v_{8}+v_{9}\\ \frac{dRC6}{dt}=v_{13}-v_{14} & \frac{dMCM_{N}}{dt}=v_{15}+v_{11}+v_{9}-v_{16}\\ \frac{dRC7}{dt}=v_{14}-v_{17} & \frac{dMCM\cdot Cdt1_{N}}{dt}=v_{6}-v_{9}-v_{7} \end{array}$$

The remaining state variables are constrained by the following conservations:

(2) $$\begin{array}{l} \text{RC1 }={\text{ RC}}_{\text{Total}}-\text{ RC2 }-\text{ RC3 }-\text{ RC4 }-\text{ RC5 }-\text{ RC6 }–\text{ RC}7\\ {\text{CDT}1}_{\text{C}}={\text{ CDT}1}_{\text{Total}}-{\text{ CDT}1}_{\text{N}}\\ {\text{MCM}}_{\text{C}}={\text{ MCM}}_{\text{Total}}-{\text{ MCM}}_{\text{N}}-\text{ RC3 }-\text{ RC4 }-\text{ RC5 }-\text{ RC6 }-\text{ FORK }-\text{ MCM}\cdot \text{CDT}1\\ {\text{CDC}45}_{\text{N}}={\text{ CDC}45}_{\text{Total}}-\text{ RC6 }-\text{ FORK} \end{array}$$

where RC_Total_, CDT1_Total_, MCM_Total_, and CDC45_Total_ are the fixed total number of origins, Cdt1 molecules, Mcm2-7 complexes, and Cdc45 dimers, respectively. These four factors have been shown to be present at constant levels throughout the cell cycle [7,61-64]. The value for RC_Total_ used in our model is 332, as described in [65].

### System inputs

The biological network responsible for the initiation of DNA replication does not oscillate autonomously; it displays periodic behaviour when driven by periodic signals from the cell cycle. Likewise, our model displays oscillations only when driven by periodic forcing input. In order to facilitate the combination of our model with the cell cycle model of Chen et al. [45], we used the simulated profiles of Clb5 and Cdc20 from their model as periodic inputs to ours. Cdc20 mediates the degradation of Dbf4 (reaction v_4_). Clb5 is responsible for Cdc6 degradation (v_2_), loading of Cdc45 (v_13_), nuclear export of free MCM (v_16_) and Cdt1 (v_8_), and phosphorylation of Orc2 and Orc6 (v_17_). We converted the time-varying profiles of Clb5 from the Chen et al. [45] model to molecules-per-cell units using the genome-wide GFP tagging and localization experiments described in [66,67]. The profile of Cdc20 was similarly obtained by scaling to cellular abundance levels reported in another study – while Cdc20 has been determined to peak at 2200 copies in a haploid cell, the functional APC^Cdc20^ level can be estimated by considering the APC cyclosome subunit Cdc27 [68,69]. This was reported in different studies to be 593 mol/cell in an asynchronous population [67] and at its maximal value of 750 mol/cell in metaphase [68].

### Data acquisition

**Figure 2:**
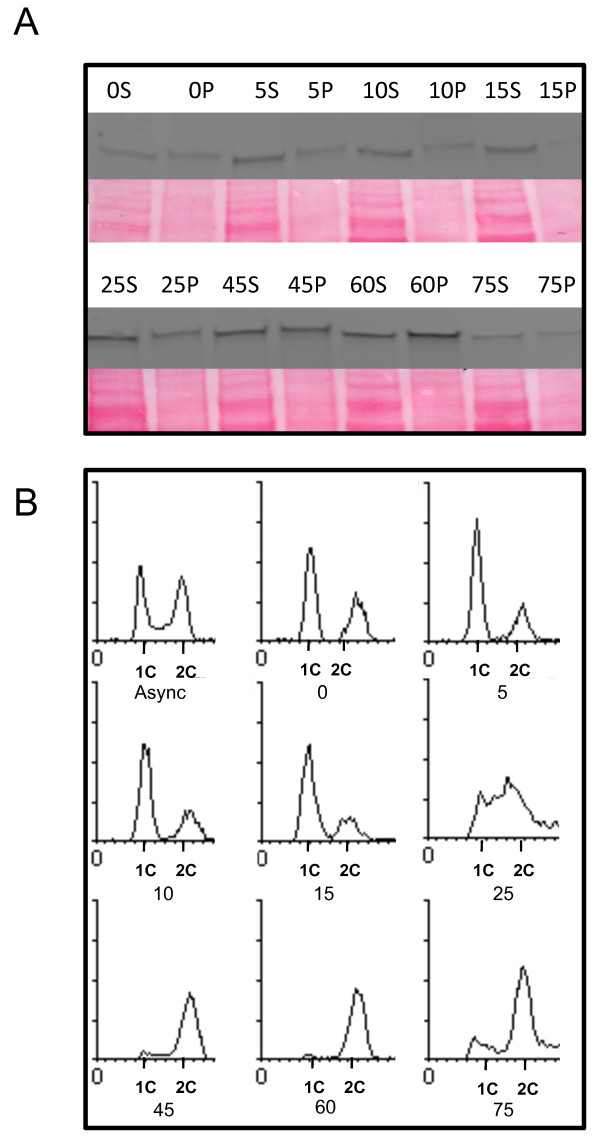
**Example of *in vivo* timecourse experiment.** (**A**) Western blot probed with α-Myc antibody to detect the Cdc6-Myc fusion protein. The corresponding Ponceau-S membrane stains are shown; these serve as loading controls to which densitometric readings were normalized. The labels indicate the time (min) elapsed since release from α-factor; S and P denote the supernatant (soluble protein) and pellet (DNA-bound) fractions, respectively. (**B**) FACS analysis of the samples described in **A**, along with an asynchronous culture sample (Async) prior to α-factor arrest.

**Figure 3:**
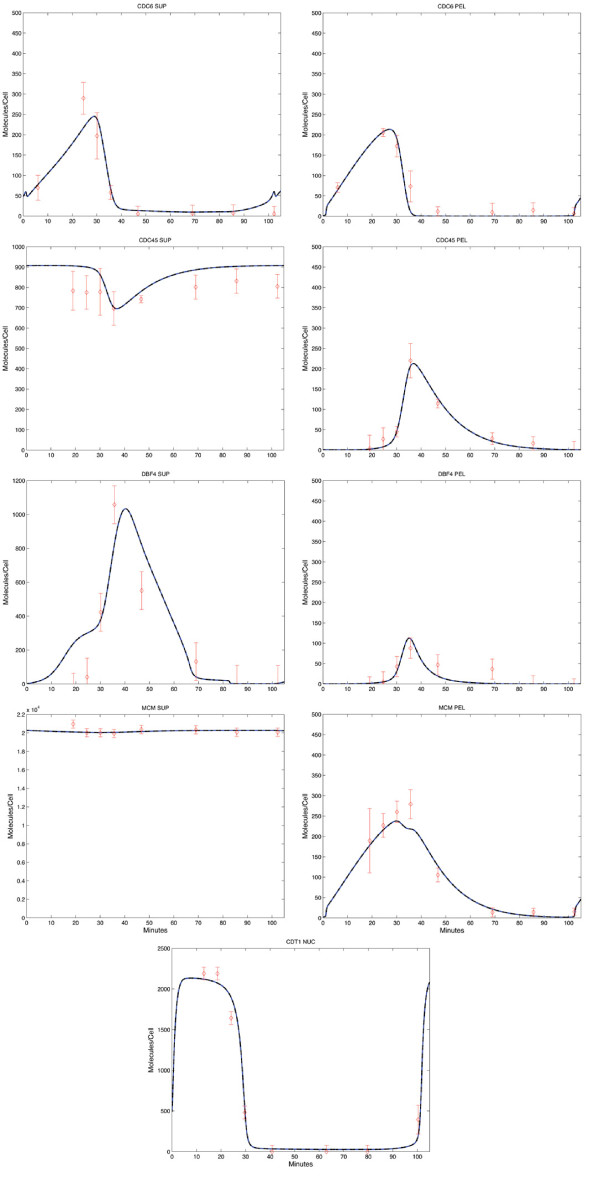
**Model-generated best fits.** Blue lines represent model simulation; red diamonds represent *in vivo* data points. PEL indicates a chromatin-bound species (pellet); SUP indicates non-chromatin bound (supernatant); NUC indicates nuclear fraction. The error bars indicate the variance calculated from triplicate experiments. Since the Cdt1 and Dbf4 data [7] and [71], respectively was not reported with variance values, we assigned values to these factors equal to the variance from the corresponding time-point for Cdc45, as these have similar abundances compared to other proteins in the model. The observed quantities correspond to the model state variables as follows: CDC6PEL = RC2 + RC3, CDC6SUP = CDC6_N_, CDC45PEL = RC6 + FORK, CDC45SUP = CDC45_N_, DBF4PEL = RC5 + RC6, DBF4SUP = DBF4_N_, MCMPEL = RC3 + RC4 + RC5 + RC6 + FORK, MCMSUP = MCM_N_ + MCM·CDT1_N_ + MCM_C_, and CDT1NUC = CDT1_N_ + MCM·CDT1_N_. One MCM molecule represents two MCM hexamers. Similarly one molecule of Cdc45 represents two individual such proteins.

Data for Cdc45 and Cdc6 levels were obtained from individual isogenic strains in which the open reading frame of the corresponding gene was fused to a sequence encoding a 13Myc epitope tag [70]. In Figure 2, a representative western blot for Cdc45-Myc is shown (panel A), with the corresponding FACS analysis (panel B). The levels of Mcm2 were determined using an anti-Mcm2 antibody. In each of our time course experiments, cells were first arrested in late G1 phase with the mating pheromone α-factor and then released synchronously into the cell cycle, as described in Methods. From the literature, we used time course data for chromatin-bound and soluble Dbf4 and Mcm2 (to supplement our *in vivo* data) from Pasero et al. [71] and quantitation of the nuclear fraction of Cdt1 from Tanaka and Diffley [7]. In order to convert relative measures of protein abundance to molecule-per-cell numbers, we used scaling factors obtained from the database provided by Ghaemmaghami et al. [67]. The data is shown along with a best-fit simulation in Figure 3. Raw timecourse data can be found in Additional files 1,2 and 3. Our calculation of molecule-per-cell estimates is demonstrated in Additional file 4. The fits in Figure 3 represent our best solution to a trade-off between quality of fit and model complexity. We explored the effect of adding additional species and parameters. These additional features could, in some cases, provide minor improvements to the fit, but our confidence in the parameter estimates (discussed in the following section) suffered as the complexity of the model grew.

While our model reports proteins and complex concentrations in absolute units of molecules/cell, our accuracy regarding these values is limited to by the literature-reported cellular abundances for the various protein factors. We have used discretion when inconsistencies arise, choosing the reported values that are best justified by multiple studies. The network dynamics are based on the relative protein abundances over the time-course (indeed, many modelling efforts describe concentration changes in arbitrary units). Consequently, changes in the global protein level for a particular factor do not affect the dynamics; instead, they impact our predictions of absolute molecule/cell counts and parameter values. This is an important consideration regarding the conversion of densitometry readings to absolute values, as the overall levels are ultimately determined by a literature-derived scaling factor. Future experiments will undoubtedly result in improved estimates of protein abundances. These can be easily incorporated into the model by scaling the parameter values accordingly (with no direct impact on system dynamics).

### Parameter calibration

The model parameters were calibrated using a weighted least-squares comparison with the data described above. We used a combination of global optimization (adaptive simulated annealing) and local search (Nelder-Mead simplex method) to find the best-fit parameter set shown in Table 2. The table also shows the percent error associated with each parameter estimate. The percent error is the relative size of a 95% confidence interval for the estimate, calculated via the Fisher information matrix and the Cramer-Rao bound [72]. The percentage errors show that some parameters are estimated with high confidence while others are represented with less accuracy.

**Table 2:** Optimal Values of Parameters Used to Describe the Network

**Description**	**Parameter**	**Value**	**Units**	**% Error**
Cdc6 production	k_1_	15.982	(Mol./cell) x min^-1^	8.86
Cdc6 degradation	k_2_	0.001	(Mol./cell)^-1^ x min^-1^	22.76
Dbf4 production	k_3_	1368.220	(Mol./cell) x min^-1^	17.18
Dbf4 degradation	k_4_	2.440	(Mol./cell)^-1^ x min^-1^	17.82
Cdc6 association with ORC	k_5_	0.016	(Mol./cell)^-1^ x min^-1^	30.86
Cdc6 dissociation from ORC	k_5r_	675.422	min^-1^	861.23
MCM-Cdt1 import	k_6_	1.015	(Mol./cell)^-1^ x min^-1^	24.88
MCM loading	k_7_	275.675	(Mol./cell)^-1^ x min^-1^	827.56
Cdt1 export	k_8_	1.732	(Mol./cell)^-1^ x min^-1^	41.81
Dissociation of MCM·Cdt1	k_9_	100.881	min^-1^	39.84
Re-association of MCM·Cdt1	k_9r_	1042.739	(Mol./cell)^-1^ x min^-1^	41.98
Dissociation of Cdc6 from RC3	k_10_	936.745	min^-1^	32.07
Re-association of Cdc6 with RC3	k_10r_	352.504	(Mol./cell)^-1^ x min^-1^	29.39
Unloading of MCM from RC4	k_11_	885.147	min^-1^	29.61
Dbf4 association with RC4	k_12_	0.568	(Mol./cell)^-1^ x min^-1^	38.91
Dbf4 dissociation with RC4	k_12r_	192.628	min^-1^	54.52
Association of Cdc45 with RC5	k_13_	0.528	(Mol./cell)^-1^ x min^-1^	54.16
Fork Firing	k_14_	0.237	min^-1^	30.08
Fork disassembly	k_15_	0.097	min^-1^	16.52
MCM export	k_16_	3.196	min^-1^	41.82
Phosphorylation of ORC	k_17_	13.313	min^-1^	239.26
Dephosphorylation of ORC	k_18_	2.497	Mol./cell	43.63
Michealis constant for import of MCM	KM_1_	195.302	Mol./cell	2123.53
Michealis constant for association of Cdc45	KM_2_	8.248	Mol./cell	2094.64

These values were used to solve the ODEs in our consensus model. The percentage error for each parameter is indicated.

Parameter values that were well constrained by the data include those specifying the rates of production, degradation and association of Cdc6 (k_1_, k_2_, k_5_) and Dbf4 (k_3_, k_4_, k_12_) as well as the rate of origin firing (k_14_). This reflects the strong reliability of our data for these two protein factors as well as for the proteins that form the replication complex (RC6) that gives rise to active forks.

Parameters values in which we have low confidence include those that govern the loading of MCM by Cdt1 (v_7_), Cdc6 dissociation from RC3 (k_5r_), and the phosphorylation of ORC (k_17_). The reversible dissociation of Cdc6 is needed to accurately fit the data and there is no evidence suggesting that ORC-Cdc6 binding is irreversible. Nevertheless, it is clear that experimental observations specific to this process are required to more precisely estimate this parameter value. The reaction whereby the MCM·Cdt1 species loads the MCM complex (v_7_) is extremely transient [10]. Provided parameter k_7_ is sufficiently large, the kinetics of this reaction will be rapid enough to fit the data. Consequently, the data cannot support a precise estimate of the parameter value. This observation suggests that MCM loading is an extremely rapid biochemical step in pre-RC assembly. It may point to a role for Cdt1 in repeatedly targeting MCM complexes to origins throughout G1. Such a phenomenon is consistent with the requirement for a dynamic loading mechanism that ensures pre-RC fidelity up until the G1/S transition. Finally, the phosphorylation of ORC (characterized by k_17_) contributes to the prevention of repeated origin firing. However, this mechanism has not been well characterized, and our data is unable to accurately constrain the specifics of this process.

The kinetic rates in this network have not been the subject of prior experiments, but previous reports of protein half-lives are consistent with our predicted parameter values. Drury et al. [31] estimated that Cdc6 is reduced below the point of detection within 5 minutes of S-phase entry, corresponding to a half-life no longer than 1.5 min. Similarly, Cheng et al. [20] reported that Dbf4 is reduced below visible levels within 10 minutes by the APC-dependent pathway, indicating a half-life no longer than 3 min. Our model-based predictions of degradation rates correspond to half-lives of 1 min. and 2.5 min. for Cdc6 and Dbf4 respectively, in good agreement with these earlier findings.

**Figure 4:**
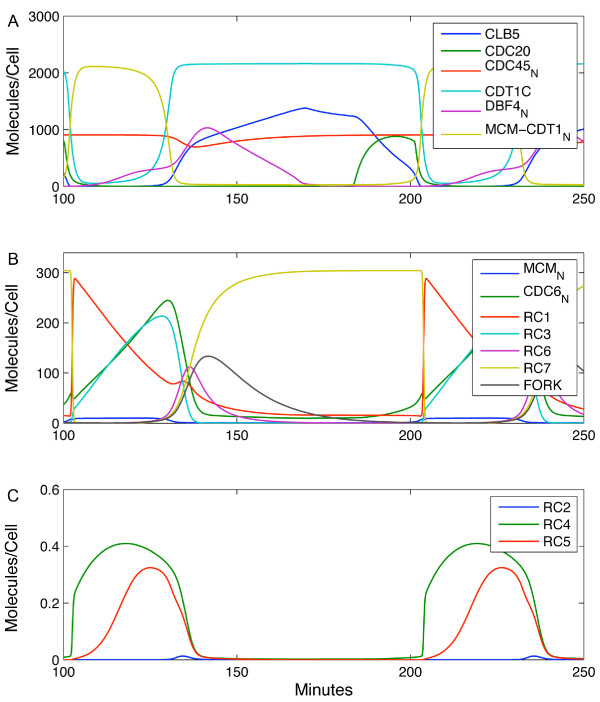
**Protein concentration profiles simulated by the model.** Panel (**A**) includes the inputs from the Chen et al. [45] model used to drive our network (Clb5 and Cdc20), scaled from arbitrary units to molecules/cell. Included are the behaviours of various protein factors within our model. Additional factors, replication complexes (RCs) as well as the FORK species are shown in panel (**B**). The transient RC species (RC2, RC4 and RC5) are shown in panel (**C**).

Figure 4 shows the simulated model behaviour for the best-fit parameter set. Some replication complex species – RC2, RC4 and RC5 – are extremely transient. Their low levels of abundance are shown separately from other RCs, on an appropriate scale. Simulations were carried out in Matlab (code available from the authors upon request).

### Perturbations

**Figure 5:**
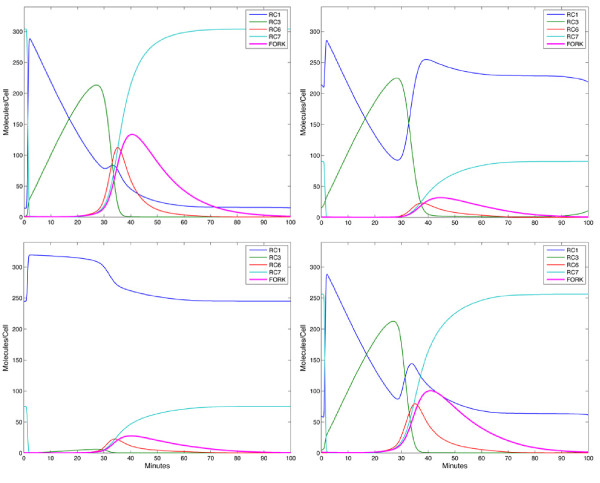
** *In silico* Simulations of Perturbations:** (**A**) Wild-type behaviour. (**B**) Expression of Dbf4 reduced to 10% of nominal. (**C**) Expression of Cdc6 reduced to 10% of nominal. (**D**) Total abundance of Cdt1 reduced to 10% of nominal. Perturbations of Cdc6 and Dbf4 had a significant impact on replicative efficiency, as evidence by a reduced abundance of activated replication forks (FORK). In contrast, a similar reduction in Cdt1 levels had much less impact.

Our initial explorations of the model revealed that the network’s behaviour is particularly sensitive to the abundance of Dbf4 and Cdc6 and relatively insensitive to the level of Cdt1. We investigated the effects of perturbations by simulating reductions in Dbf4, Cdt1 and Cdc6 (Figure 5) in our model. When the Cdc6 production rate (v_1_) was reduced to 10% of its nominal (wild-type) value, persistence of the RC1 complex was observed. Similarly, when the Dbf4 production rate (v_3_) is reduced by the same relative amount, an accumulation of RC3 occurs. In both cases, the perturbation interferes with pre-initiation complex assembly and blocks the system at the nearest previous persistent RC state; RC4 is not persistent since the unloading of MCM causes a rapid transition back to RC1. It is worth noting that because MCM can dissociate from ORC (v_22_), RC4 represents a complex containing MCMs that will be functionally incorporated into replication forks as opposed to those that loosely associate with origins. Because the timing of our model is fixed, the various state concentrations (RC levels) indicate the progression from licensing to firing. A reduction in the FORK species compared to the wild-type case suggests a slow-down in S-phase because fewer origins are firing within the prescribed time. Using the peak abundance of the FORK species as a measure of replicative efficiency, we saw significant reductions in both simulated knock-downs (by 68% for Dbf4 and 73% for Cdc6, Figure 5B, C). Conversely when we simulated the reduction of Cdt1 abundance to 10% of nominal values, origin firing was only reduced by 23%, suggesting that the network is relatively refractory to depletion of Cdt1 (Figure 5D). Additional file 5 shows the levels of the various model components for each perturbation.

**Figure 6:**
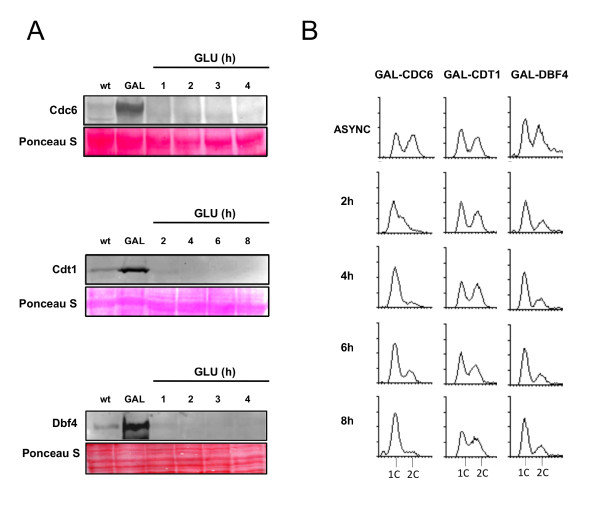
**Experimental Investigation of Protein Depletion Below Normal Endogenous Levels for Dbf4, Cdc6 and Cdt1.** (**A**) Asynchronous cultures of *GAL1-CDC6* (DY-139), *GAL1-CDT1* (DY-140), *GAL1-DBF4* (DY-255) and their wild-type counterparts DY-142, DY-143 and DY-256, respectively, were grown to 10^6^ cells/ml in galactose (GAL) medium, washed and resuspended in glucose (GLU) medium. Whole-cell extracts were prepared from culture aliquots taken prior and post shift from galactose to glucose with indicated time points corresponding to time in glucose medium. HA-tagged Cdc6 and HA-tagged Cdt1 were detected using an anti-HA antibody (Sigma) and a fluorescent secondary antibody (Invitrogen). Ponceau S staining of the region detected by the blot to judge loading of whole-cell extracts is also shown. (**B**) FACS analysis of culture aliquots from either asynchronous (Async) cultures, or at the indicated times after cell resuspension in glucose medium.

To investigate the accuracy of these mathematical predictions, we carried out corresponding wet lab depletion experiments. Reducing Dbf4 or Cdc6 concentrations in yeast cells to roughly 90% below normal endogenous levels resulted in a rapid G1 phase arrest, evident after 2 hours of depletion, as judged by FACS analysis indicating the accumulation of cells with 1 C (unreplicated) DNA content (Figure 6). In contrast, a corresponding depletion of Cdt1 had no appreciable effect, and DNA replication defects were still only minimally evident after 8 hours of further reduction. Thus, our *in silico* simulations using our nominal parameter set were predictive of *in vivo* perturbations. These experiments were used to validate the model; they were not used for calibration.

The insensitivity to perturbations in Cdt1 levels is consistent with its apparent excess relative to origins [67], although the number of Cdt1 molecules that act at each origin has not yet been characterized. Moreover, the mechanism by which Cdt1 aids in recruiting the helicase molecules to pre-RCs is extremely transient [10].

While many factors are limiting, the system appears to be highly sensitive to the levels of Cdc6. Due to its low abundance relative to MCM and Cdt1, even a moderate depletion of Cdc6 significantly alters the dynamics of pre-RC loading. The same is true for Dbf4, although in this case its role in activation of the Cdc7 kinase renders the system highly sensitive to its concentration; firing cannot occur without the Dbf4-Cdc7 complex. Since Dbf4 is, like Cdc6, limiting, flow through the network is blocked when the kinase does not reach a threshold level. Additionally at limiting levels, the number of replication forks produced by our model is significantly reduced, consistent with *in vivo* reports from the literature showing a lengthening of S-phase [15].

**Figure 7:**
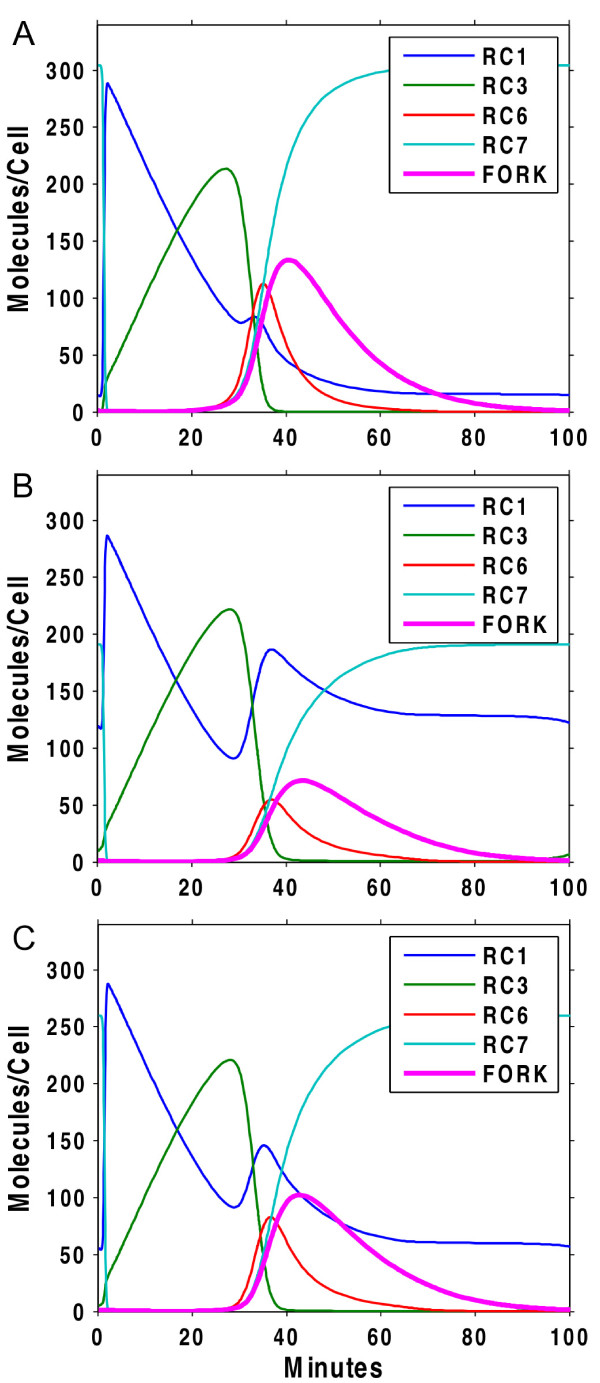
** *In silico* perturbations to the consensus model agree with reported *in vivo* cell cycle defects.** (**A**) Wildtype behaviour. (**B**) Reduction of the rate of Dbf4-MCM association (k_12_) to 50% of the nominal value. (**C**) Reduction of the rate of Cdc45 interaction with the pre-RC (k_13_) to 50% of the nominal value. In both cases, a decrease in the abundance of the FORK species indicates a defect in DNA replication.

Further validation of our model comes from comparison with additional *in vivo* experiments reported in the literature. Jones et al. [73] showed that the interaction between the MCM complex and Dbf4 was reduced to half its wild-type level when a Dbf4 domain that binds Mcm2 was mutated, impairing S-phase progression. We mimicked this effect by reducing the rate of association of Dbf4 with RC4 (k_12_) by 50%, leading to a similar result (compare Figure 7 panels A and B). Similarly, Zou et al. [74] reported that the *cdc45-1* mutant shows an aberrant growth phenotype at the non-permissive temperature. This is thought to be due to a disruption of Cdc45’s ability to interact with MCM and ORC (RC6). As shown in Figure 7C, by reducing the rate of Cdc45 interaction with RC6 (k_13_) by 50%, a marked reduction in the peak abundance of the FORK species results, indicative of a slower S-phase, as observed when the mutant was grown at the non-permissive temperature. The actual reduction in Cdc45’s association with the pre-RC due to conformational changes in the mutant might be even more pronounced than a 2-fold reduction. In any case, our simulation is consistent with Cdc45’s origin-initiation role being compromised by impairing its ability to interact with its ligands to form the CMG complex.

### Linking our DNA initiation model to a previously established cell cycle model

Our model of the initiation of DNA replication only displays oscillatory behaviour when forced with periodic signals from the cell cycle. By choosing to incorporate signals that correspond to species in the cell cycle model of Chen et al. [45], we were able to merge the two models in a straightforward manner. In the Chen model, the initiation of DNA replication is represented by a single lumped state variable, called ORI. At the beginning of the cell cycle, ORI has value zero. Its rate of growth depends linearly on Clb5. When it reaches a threshold value, DNA synthesis is presumed to have begun, and triggers an increase in the value of the parameter k_mad2_ (activity level of the Mad2 protein) leading to an inactivation of Cdc20, which is required for mitotic exit. This Mad2-dependent inhibition of Cdc20 represents the spindle assembly checkpoint [75], ensuring that cells with replicated DNA do not complete mitosis without properly aligning the chromosomes. When chromosomes have properly aligned on the metaphase plane k_mad2_ drops and Cdc20 promoted exit from mitosis. In our model, the level of DNA synthesis is represented by the FORK species. To merge the two models, we removed the ORI state from the Chen model, and instead used the FORK species to trigger the change in k_mad2_, as detailed in Methods.

Besides “closing the loop” between the two models by incorporating two-way inter-model signalling (involving Clb5, Cdc20, and FORK), we had to deal with a single shared species: both models describe the dynamics of Cdc6. We arrived at a merged description of Cdc6 behaviour by incorporating the dynamics of recognition complex association and dissociation into the Chen model’s formulation of Cdc6 behaviour (details are described in Methods). The resulting combined model behaves only marginally differently from either model in isolation, as shown in Additional file 6: Figure S6 and Additional file 7: Figure S7).

### Replication metrics of Brümmer et al

The recently published model of Brümmer et al. [54] also describes the network responsible for the initiation of DNA replication. The 51 free parameters of that model were chosen by a combination of fitting and optimization. The authors used literature-derived data to fix 28 of the kinetic parameters. The remaining 23 free parameters were not fit to data, but were selected through a procedure that optimized the coherence of origin firing and minimized re-replication (selected as hypothetical goals of evolutionary ‘design’). While it is impossible to assess the accuracy of the parameter values obtained from this procedure, the resulting idealized model provides a useful starting point for examining how the network structure constrains the system behaviour.

The model of Brümmer et al. focuses on early origin firing and so represents the mechanics of firing at the start of S-phase. In contrast, our model describes firing dynamics throughout S-phase in order to fit into the broader context of the cell cycle [76-79]. The parameter set driving our system is not filtered to retain only those that produce replication dynamics consistent with coherent firing just at the G1/S boundary. Rather, the parameters are specified by the actual cellular concentrations of the active protein factors generating replication forks. While both models incorporate the important role of CDK, Brümmer et al. emphasize the multi-site phosphorylations of several factors involved in mechanisms that minimize potential re-replication. To this end, Brümmer et al. employed a metric to assess re-replication. Their idealized model exhibits 0.0028 re-replication events per cycle. Applying the same measure to our bottom-up model yields 0.36 re-replication events per cycle (although that can readily be reduced by modifying our parameters from their best-fit values). The near-zero value obtained by Brümmer et al. is close to their idealized target of zero. Both estimates are consistent with the belief that re-replication occurs in wildtype cells, but at an extremely low rate [79]. Because the nature of the dephosphylation of ORC (RC7→RC1 transition) remains uncharacterized, we use a conservative estimate of the number of ORC phosphorylation sites. Increasing this number by twofold, consistent with the number of CDK target residues on ORC [32] reduces re-replication to a value on the same order of magnitude as Brümmer’s value. Thus, both models effectively deal with representing control and prevention of rereplication.

## Conclusions

While our model provides a sound description of the initiation of DNA replication, a number of aspects of the network remain unresolved: for example, the kinetics of MCM loading, the mechanism by which CDK phosphorylates ORC, and the details of the association of the GINS complex, Sld2, and Sld3. While modeling Cdc45 captures the events regarding CMG formation at origins, being its limiting factor, a future version of the model could better distinguish the initial Cdc45 association at origins from subsequent CMG formation. While this has minimal effect on our network dynamics and no effect on our blocking of re-replication, it would provide a better resolution of events at origins just prior to the G1/S transition. Incorporating timecourse experiment data for levels of a GINS complex member would aid in this analysis. Nevertheless, the assumptions made allow us to approximate the aforementioned processes, simplifying the network without losing information about system behaviour at the level we intend to model. With our nominal parameter set, we observe the system to behave as the ordered accumulation of proteins forming a loading complex at origins throughout the genome. Activation by increasing S-CDK levels and the concentration of Dbf4 (regulating kinase activity of Cdc7) increase linearly the number of replication forks established as a result of successive forward transition of the various replication complexes (RCs). It should be noted that as is found *in vivo*, not all origins fire as a consequence of being furnished with MCM-containing pre-RCs. Replication is maximal at the G1/S transition, but continues into S-phase as origin firing is temporally spaced. This is thought to ensure sufficient time to address any defects in replication and is mediated by the limiting nature of one of the initiation activators, DDK (reviewed in [55]). Our *in vivo* perturbation of Dbf4 levels reproduces this consequence and points to other system observations: Cdc6 levels are intimately controlled by CDK levels to avoid re-replication, however this mechanism is tightly regulated such that Clb5 levels rising too soon would prevent the assembly of the pre-RC in G1, a feature of the system well documented. Additionally, Cdt1 appears to act catalytically rather than stoichiometrically given the system is relatively impervious to a reduction of this factor to 10 % of its wildtype level. This might play into its role in chaperoning Mcm2-7 hexamers to origins, where they are loaded subsequently leading to the release of Cdt1, which may then be recycled to mediate the loading of other MCMs. This aspect of the system has not yet been investigated experimentally and would be of future interest.

Many human orthologs of the yeast proteins described in our network have been associated with cellular pathologies. Our model is specific to the replication machinery in budding yeast, but the mechanisms driving this process are highly conserved throughout Eukarya. Efforts to develop an analogous model in mammalian cells would be useful in understanding and dissecting cell proliferation in humans. A number of models of the mammalian cell cycle have been proposed [80-84]. Incorrect pre-RC formation has been linked to impaired DNA damage repair pathways in humans [85], while both Orc6 [86] and members of the Mcm2-7 complex (reviewed in [87]) have been shown to be reliable cancer biomarkers. Recent work by Bicknell et al. [88,89] has shown that point mutations in the human ORC1, ORC4, ORC6, CDT1 and CDC6 genes are associated with Meier-Gorlin syndrome, a form of primordial dwarfism, and several of these mutations were determined to interfere with proper pre-RC formation. These findings highlight the potential utility of *in silico* mammalian models in further exploring the molecular basis of such disorders. Given that our model shows good predictive capability, it serves not only as an informational tool for yeast biology, but also as a proof of principle for higher order system models. Despite the requirement for a mammalian model to comprehensively verify specific mechanisms, the system of DNA replication initiation is conserved well enough that perturbations to proteins such as those described above can, in fact be preliminarily examined.

Although previously established replication models [51,52,54] consider the ordered timing of origin firing based on genomic replication profiles, our goal was to represent the temporal organization of origin firing as a function of the concentration of active replication species. A focus on using real protein levels as a determinant of replication dynamics is a novel approach. When used in concert with models describing genome-level origin characteristics and/or combining the findings with models exploring other cell-cycle modules, future efforts will generate a well-rounded picture of DNA replication initiation.

## Methods

### Yeast strains and transformants

Details of the strains used in this study are listed in Table 3. All strains are isogenic and derived from the wild-type strain DY-26.

**Table 3:** Strains used in the experimental protocols of this study

**Strain**	**Genotype**	**Source**
DY-26	MATa, his3Δ200, leu2Δ, met15Δ, trp1Δ63, ura3Δ	ATCC (B44733)
DY-82	MATa, his3Δ200, leu2Δ, met15Δ, trp1Δ63, ura3Δ, CDC45::CDC45-13MYC (HIS3)	This study
DY-128	MATa, his3Δ200, leu2Δ, met15Δ, trp1Δ63, ura3Δ, CDC6::CDC6-13MYC (HIS3)	This study
DY-139	MATa, his3Δ200, leu2Δ, met15Δ, trp1Δ63, ura3Δ, CDC6::Pgal1-3HA-CDC6 (TRP1)	This study
DY-140	MATa, his3Δ200, leu2Δ, met15Δ, trp1Δ63, ura3Δ, CDT1::Pgal1-3HA-CDT1 (TRP1)	This study
DY-142	MATa, his3Δ200, leu2Δ, met15Δ, trp1Δ63, ura3Δ, CDC6::CDC6-3HA (TRP1)	This study
DY-143	MATa, his3Δ200, leu2Δ, met15Δ, trp1Δ63, ura3Δ, CDT1::CDT1-3HA (TRP1)	This study
DY-255	MATa, his3Δ200, leu2Δ, met15Δ, trp1Δ63, ura3Δ, DBF4::Pgal1-3HA-DBF4 (TRP1)	This study
DY-256	MATa, his3Δ200, leu2Δ, met15Δ, trp1Δ63, ura3Δ, DBF4::DBF4-3HA (TRP1)	This study

### Cell cycle timecourse

Cells were grown in YPD medium (1% Bacto-yeast extract, 2% Bacto-peptone, 2% dextrose) to exponential phase at 30°C, washed with dH_2_0 and resuspended in fresh YPD at a concentration of 1 x 10^7^ cells/ml. Cultures were subsequently arrested in late G1 phase with the addition of 5 μg/ml alpha-factor peptide (Sigma-Aldrich) for 2.5 hours. Cells were monitored for G1 phase arrest through microscope observation of the percentage of unbudded cells. Approximately 1.5 × 10^7^ cells were collected and treated with 0.1% sodium azide then kept on ice at 4°C. The remainder of the arrested culture was then centrifuged at 200 *g* and the pellet was washed twice with dH_2_0. The cells were all *BAR*^*+*^ and thus secrete the Bar1 protein, which degrades the alpha-factor pheromone used. Medium was collected and saved during centrifugation of the original logarithmic culture containing this protein and was subsequently used to resuspend the cells for the release from G1 phase. Additionally, Pronase E (Sigma-Aldrich), an enzyme that also hydrolyzes alpha-factor, was added at a concentration of 10 μg/ml to facilitate a synchronous release. Roughly the same number of cells collected in the G1-arrested samples were collected at the other time points following release and similarly treated with sodium azide and kept on ice until the completion of the time course.

### Fluorescence activated cell sorting (FACS)

To assess cell synchrony, 1.5 × 10^6^ cells were removed from each time point sample. They were immediately centrifuged, re-suspended in 1 ml of ice-cold 70% ethanol and stored overnight at 4°C. Cells were then re-suspended in 500 μl of 50 mM Tris-HCl pH 8 containing 10 mg/ml of RNase A and incubated for 2 hours at 37°C. This was followed by centrifugation and re-suspension in 500 μl 50 mM Tris-HCl 7.5 with 2 mg/ml Proteinase K. Incubation at 50°C for an hour was performed prior to final resuspension in 100 μl FACS buffer (200 mM Tris-HCl 7.5, 200 mM NaCl and 78 mM MgCl_2_). Cells were stained with SYTOX Green dye (5 μM; Molecular Probes) for at least one hour and then analyzed using a Becton-Dickinson FACScan.

### Chromatin fractionation

Chromatin fractionation was performed as described by Semple et al. [8] with some modifications. Approximately 1 × 10^7^ cells collected from each time point were incubated in 7.5 ml pre- spheroplasting buffer (100 mM EDTA-KOH pH 8, 10 mM DTT), after washing once with dH_2_O. They were then incubated at 30°C for 10 min with gentle shaking. Cells were centrifuged and re-suspended in 7.5 ml spheroplasting buffer (1 X YPD, 1.1 M sorbitol) containing 0.5 mg/ml Zymolyase 20 T (Seikagaku Corp., Japan) and 0.1 mg/ml Oxalyticase (Sigma), followed by shaking at 30°C for 30-45 min with gentle mixing. Cells were then washed once with 20 ml spheroplasting buffer containing 0.5 mM PMSF followed by resuspension in 1 ml ice-cold wash buffer (5 mM Tris–HCl pH 7.4, 20 mM KCl, 2 mM EDTA-KOH pH 7.4, 1 M sorbitol, 1% thiodiglycol, 125 mM spermidine, 50 mM spermine). Wash, Breakage and Lysis buffers all contained 1 tablet/10 ml of EDTA-free protease inhibitors (Roche) and were supplemented with 0.5 mM PMSF. Cells were centrifuged at 400 *g* for 1 min at 4°C, washed twice with 1 ml of Wash buffer and then re-suspended in 800 μl of Breakage buffer (5 mM Tris–HCl pH 7.4, 20 mM KCl, 2 mM EDTA-KOH pH 7.4, 0.4 M sorbitol, 1% thiodiglycol, 125 mM spermidine, 50 mM spermine). To these cells, 1 ml of Lysis buffer (Breakage buffer supplemented with 1% Triton X-100) was added and after repeated inversion (until the solution turned clear), cells were pelleted at 16,000 *g* for 10 min. This separated the proteins bound to the chromatin (residing in the pellet, referred to as PEL) from those solubilized in the non-chromatin fraction (supernatant or SUP). After removal of the supernatant, an additional 1 min spin at 16,000 *g* was performed to isolate any residual supernatant and the pellet was re-suspended in 100 μl Breakage buffer. MgCl_2_ (5 mM) and DNase I (2 μg/ml) were added to the PEL fractions to solubilize the chromatin and associated proteins. After 10 min, the reaction was quenched with the addition of 2 μl 0.5 M EDTA. 10 μl of each SUP and PEL sample were collected for protein quantification. To the remaining of each sample, ½ volume of loading buffer (270 mM DTT, 9.9% SDS, 26% Glycerol and 10% Bromophenol blue) was added. Samples were subsequently boiled and equal concentrations of total protein were loaded on 7.5% SDS-PAGE gels. For an equal volume, PEL samples were 20-fold more concentrated than SUP samples due to the fact that approximately 5% of proteins are chromatin bound.

### Western blotting and densitometry

Chromatin fractionation samples were assayed for protein concentration using the Bradford assay (Biorad). Equal amounts of protein were loaded into each lane of a 10% SDS-polyacrylamide gel for each set of time course samples. Following transfer, nitrocellulose membranes were stained with Ponceau S dye (Sigma). Membranes were destained using 1 X TEN buffer for 10 min. Mouse anti-Myc (Sigma, 1:5000) and Alexa Fluor 488 goat anti-mouse IgG (Invitrogen, 1:3000) antibodies were used to detect Myc-tagged Cdc45 and Cdc6. Anti-Mcm2 antibody (yN-19 goat polyclonal, Santa Cruz, 1:500) along with Alexa Fluor 488 donkey anti-goat IgG (Invitrogen, 1:3000) antibodies were used to detect Mcm2. Blots were also probed with TAT1 antibody (gift from the Gull lab) for visualizing α-tubulin, which should be exclusive to SUP samples as it does not associate with chromatin. Blots were incubated in primary and secondary antibodies for 2 h each, proceeding 4 h of blocking in 5% skim milk. Between blocking and each antibody treatment, blots were washed for 2 × 10 min with 1 x TEN + 0.05% Tween-20. A Typhoon 9410 scanner (GE healthcare) was used to analyze the blots. Densitometry readings were performed using Image Quant TL software (BD) and were normalized to total protein concentration as judged by the Ponceau S stain and/or tubulin band intensity. The same protocol for Western blotting was used in perturbation experiments involving HA-tagged strains. In this case, mouse anti-HA (Sigma, 1:5000) and Alexa Fluor 488 goat anti-mouse IgG (Invitrogen, 1:3000) antibodies were used to determine levels of Cdc6, Cdt1 and Dbf4.

Determination of *in vivo* cellular concentrations for each factor was performed for each time point. Normalization of densitometry readings was carried out by averaging the means of all SUP values from each set of experiments for a given protein. This average was divided by the means of the SUPs for each trial to give a scaling factor (S1) for each trial. Each SUP value for a given trial was then multiplied by its scaling factor (S1, S2 or S3). The same procedure was applied to all PEL sample values. In general, when a protein is in the chromatin fraction it is DNA-bound. To correct for non-specific DNA-binding, we determined, for each protein, a background level of non-specific binding corresponding to the observed abundance from a time-point at which the factor is known to be absent from origins. To obtain densitometric values, the program Imgage Quant™ was used to analyze blots scanned by a Typhoon™ 9400 imager (both GE Healthcare). To convert the densitometry measures to molecules/cell concentrations, we determined a scaling factor for each protein from the molecule counts reported in [67]. For each experiment, we averaged the total densitometry measure (SUP plus PEL) across the time-points to arrive at an averaged asynchronous densitometry reading (weighted according to the time contribution of each sample to a 90 minute cycle), which was then compared to the database to arrive at a scaling factor. We could not follow this procedure for Cdc6 or Dbf4, since they are not included in the Ghaemmmaghami et al. [67] database. A scaling factor was determined for these proteins by determining the relative abundance between Cdc45, Cdc6 and Dbf4 using similarly tagged asynchronous cultures. Whole cell extract levels of Cdc45-Myc and Cdc6-Myc were analysed by Western blotting and densitometry to yield an approximately 3:1 ratio of Cdc45 to Cdc6 copies per cell. Similarly, Cdc6-HA and Dbf4-HA were compared. This analysis allowed us to arrive at an asynchronous value of 576 molecules/cell for Cdc6 (Cdc6_Total_ = 576), i.e. one third of the value reported for the concentration of Cdc45, i.e. 1730 copies/cell according to [67] (Cdc45_Total_ = 1730). Similarly, the concentration of Dbf4 in an asynchronous population was 270 molecules/cell (Dbf4_Total_ = 270). The copy/cell number used for Mcm2 was 40,000 [58] while that for Cdt1 was 2190 [67] (Cdt1_Total_ = 2190). As an example of the agreement of our simulated values with literature-observed origin stoichiometry, Mcm2 (representing the MCM complex) was present at levels that were consistent with having two MCM complexes bound to each origin. This reflects the head to head placement of the heterododecamer at the origin. Once firing occurs, two forks are produced, illustrated in our model as one Mcm2-7·Cdc45 species molecule being generated (each FORK in the model represents a pair of these complexes). The level of chromatin-associated MCM protein that we obtained corresponds to roughly 300 origins being bound in this manner, which is the range of the number of origins that are reported to *potentially* fire per cell per cell cycle according to various global origin characterization studies [65,90-92].

The values used to assign copies/cell numbers were taken from the best literature source available. The number for Mcm proteins in the GFP-tag database [67] differ by orders of magnitude from the number that has been reported by [58]. The latter is widely accepted, and so this discrepancy cannot be ignored. While similar inconsistencies might exist for other proteins in our model, where possible, we have carefully evaluated whether the GFP-tag database number gives a plausible cellular abundance through comparison with other reported values. For example, levels for Cdc45 in [67] are consistent with the value reported by [56], while no value has been reported for Cdt1 except for that in the GFP-tag database. In the event that future studies provide more accurate values for the various cellular abundances, the model can be easily revised to accommodate these changes by scaling the parameters associated with the relevant species. Indeed, it is common practice for models to be presented in terms of ‘arbitrary’ concentration units. Our use of a molecule-per-cell concentration scale allows us to make absolute predictions about species time-courses. Any future adjustments to the species levels will scale the concentrations and parameter estimates, but will have no direct bearing on the overall dynamic behaviour of the network.

### Perturbation experiments

DY-139 (*GAL1-CDC6*), DY-140 (*GAL1-CDT1*) and DY-255 (*GAL1-DBF4*) strains were grown to 1 × 10^7^ cells/ml in 2% galactose/1% raffinose medium at 30°C, centrifuged at 6000 *g* for 5 min and washed with dH_2_0. Cells were then resuspended in 2% glucose medium, maintaining the same cell concentration. Culture aliquots were removed for FACS analysis and preparation of whole cell extracts (as described in [93]), both before, and at various intervals after the switch to glucose. As references for normal endogenous protein levels, whole cell extracts were also prepared from strains expressing *DBF4* (DY-256), *CDC6* (DY-142) and *CDT1* (DY-143) from their endogenous promoters. All strains produced fusion proteins with a 3HA epitope tag to facilitate visualization via Western blotting.

### Time-varying model inputs

We collected protein data from yeast strains which were observed to have a generation time of ~90 min in log phase. In order to fit our data to the timescale of the Chen et al. [45] model, which has a period of 101.2 minutes, we scaled our experimental time-course to reflect this change in timing. This value, as described in their model, is chosen to reflect the longer cycle of daughter cells (which are smaller than mother cells in asymmetric cell division). Time-point samples were collected from an alpha factor arrest in late G1 phase, corresponding to experimental time-point T = 0. Additional samples were collected at 5, 10, 15, 30, 45, 60 and 75 min after synchronous release from the alpha factor block. We observed that our experimental timepoint T = 0 corresponds to 19 min after the beginning of G1 phase in the 101.2 min model. We arrived at this number by comparing the point at which cells entered S phase *in vivo* (~15 min after alpha factor release as determined by FACS analysis) and the corresponding start of S phase in the model.

One of the two inputs we used from the Chen model was the APC co-factor Cdc20. Its role in our model is to activate the APC, which rapidly degrades Dbf4. In the Chen model, Cdc20 serves exclusively as a signal to exit mitosis. The Cdc20 degradation rate is a function of the parameter k_mad2_, which describes the activity level of the protein Mad2, a key factor in the spindle assembly checkpoint. In order to prevent the occurrence of mitosis before replicated chromosomes have been properly aligned along the metaphase plane, the value of k_mad2_ jumps discontinuously from 0.01 to 8 once DNA replication has commenced, signified by the lumped variable ORI reaching a threshold value of 1. This ensures that Cdc20 levels are low, and so prevents premature mitosis. Later, when spindle assembly is complete, the value of k_mad2_ falls back to 0.01, allowing Cdc20 to accumulate. The signal for this event is a second lumped parameter, SPN, hitting its threshold value of 1. As a result of these discontinuous transitions in k_mad2_, the Cdc20 profile shows rather sharp shifts in behaviour. When we applied this profile as an input to our model, the Dbf4 profile spiked earlier and more abruptly than our laboratory observations; this discrepancy was not observed in the Chen model since that model does not address the influence of Cdc20 on Dbf4. To address this inconsistency, we smoothed the k_mad2_ profile to allow for a gradual decline in Mad2 activity. We chose the timing to match our Dbf4 observations. The original formulation for k_mad2_ specifies a value of 0.01 when ORI is less than 1 or SPN is greater than 1, and otherwise is equal to 8. We replaced this condition with:

(3) $$k_{mad2}=7.99*({\left(ORI/15\right)}^{\left(1/2\right)})\times 1/(1+SPN^{300})+0.01$$

The difference between the original Cdc20 profile and our modified version is shown in Additional file 8.

### Implementation of a combined model

In order to implement a combined model, we closed the loop between our model and the cell cycle model of Chen et al. [45] by eliminating the lumped species ORI and replacing it with an indication of the progress of replication from our FORK species. In this combined model we modified the formula for k_mad2_ shown above by replacing the ratio (ORI/15) with (∫FORK/500), where the integration begins at the start of the cell cycle. Since each model incorporates a description of Cdc6 dynamics, we merged them by including the dynamics of origin binding from our model with the Cdc6 dynamics of the Chen model. This resulted in good accordance between the behaviour of the replication initiation network in our model in the combined model. There are two marginal differences, neither of which affects the overall system behaviour (see Additional file 8). Firstly, due to the fact that our internal model uses a Clb5 profile generated by scaling from arbitrary units, it is nearly identical to the Chen model Clb5 profile. The Chen Clb5 profile extends farther past the 101.2 minute mark than ours resulting in a more rapid dephosphorylation of RC7 in the internal model. Thus, RC7 persists for ~5 mins longer in the combined model. Secondly, because the merged Cdc6 decreases in concentration earlier than in our model in isolation, RC1 levels stay high until fork firing occurs. Despite these differences, the essential dynamics of the system are preserved: replication fork firing follows the same pattern, with the RC7→RC1 delay having no effect on timing of firing. The behaviours of the Chen model species are not perceptibly altered by the removal of ORI and the combination of the two models (Additional file 6).

The effects of combining our models are shown to be minimal in the wildtype case. Comparing eight cell cycle mutants used to fit the Chen model in the combined model, we see the identical phenotypes (see Additional file 7 and 9). These results demonstrate that our replication initiation module is a functional replacement for the corresponding black box in Chen’s whole cell cycle model. A summary of simulated mutant phenotypes [7,55,60,73,74,94-97] is given in Additional file 9.

## Competing interests

The authors declare that they have no competing interests.

## **Authors’ contributions**

The model was conceived by GL, BI, BM and BD. Initial model implementation was carried out by PS and BI. All experimental work (except for Figure 6, RG and LD) was carried out by RG, supervised by BD. The final model implementation and analysis were done by RG and BI. The manuscript was prepared by RG. All authors read and approved the final manuscript.

## Supplementary Material

Additional file 1 **Figure S1. *In vivo* chromatin fractionation results for Cdc6 as assayed via Western blotting.** Click here for file

Additional file 2 **Figure S2. *In vivo* chromatin fractionation results for Cdc45 as assayed via Western blotting.** Click here for file

Additional file 3 **Figure S3. *In vivo* chromatin fractionation results for Mcm2 as assayed via Western blotting.** Click here for file

Additional file 4 **Table S1.Sample conversion of densitometry values to molecules per cell values for a Cdc45-myc timecourse experiment**[66]. Click here for file

Additional file 5 Figure S4. Levels of model components when Cdc6, Cdt1 or Dbf4 have been reduced to 10% of their wild-type levels. Click here for file

Additional file 6 **Figure S5. The combination of DNA replication and whole cell cycle models does not alter either’s behaviour in isolation**[45]. Click here for file

Additional file 7 **Figure S6.Mutant phenotypes reported in whole cell cycle model are unaltered in the combined model**[45]. Click here for file

Additional file 8 **Figure S7. Comparison of Cdc20 time-varying profiles as originally modeled by Chen **[24] versus our modified version [45]. Click here for file

Additional file 9 **Table S2. Summary of simulated cell cycle mutants in the internal DNA replication initiation model. Table S3.** Comparison of cell cycle mutants simulated by Chen et al. [45] and by the combined model [7,45,55,60,73,74,94-97]. Click here for file

## References

[B1] Bell SP Stillman B ATP-dependent recognition of eukaryotic origins of DNA replication by a multiprotein complex Nature 1992 357 6374 128 134 1579162 10.1038/357128a0

[B2] Rao H Stillman B The origin recognition complex interacts with a bipartite DNA binding site within yeast replicators Proc Natl Acad Sci U S A 1995 92 6 2224 2228 7892251 10.1073/pnas.92.6.2224 PMC42456

[B3] Rowley A Cocker JH Harwood J Diffley JF Initiation complex assembly at budding yeast replication origins begins with the recognition of a bipartite sequence by limiting amounts of the initiator, ORC EMBO J 1995 14 11 2631 2641 7781615 10.1002/j.1460-2075.1995.tb07261.x PMC398377

[B4] Tanaka S Umemori T Hirai K Muramatsu S Kamimura Y Araki H CDK-dependent phosphorylation of Sld2 and Sld3 initiates DNA replication in budding yeast Nature 2007 445 7125 328 332 17167415 10.1038/nature05465

[B5] Speck C Chen Z Li H Stillman B ATPase-dependent cooperative binding of ORC and Cdc6 to origin DNA Nat Struct Mol Biol 2005 12 11 965 971 16228006 10.1038/nsmb1002 PMC2952294

[B6] Speck C Stillman B Cdc6 ATPase activity regulates ORC x Cdc6 stability and the selection of specific DNA sequences as origins of DNA replication J Biol Chem 2007 282 16 11705 11714 17314092 10.1074/jbc.M700399200 PMC3033201

[B7] Tanaka S Diffley JF Interdependent nuclear accumulation of budding yeast Cdt1 and Mcm2-7 during G1 phase Nat Cell Biol 2002 4 3 198 207 11836525 10.1038/ncb757

[B8] Semple JW Da-Silva LF Jervis EJ Ah-Kee J Al-Attar H Kummer L Heikkila JJ Pasero P Duncker BP An essential role for Orc6 in DNA replication through maintenance of pre-replicative complexes EMBO J 2006 25 21 5150 5158 17053779 10.1038/sj.emboj.7601391 PMC1630405

[B9] Chen S de Vries MA Bell SP Orc6 is required for dynamic recruitment of Cdt1 during repeated Mcm2-7 loading Genes Dev 2007 21 22 2897 2907 18006685 10.1101/gad.1596807 PMC2049192

[B10] Randell JC Bowers JL Rodriguez HK Bell SP Sequential ATP hydrolysis by Cdc6 and ORC directs loading of the Mcm2-7 helicase Mol Cell 2006 21 1 29 39 16387651 10.1016/j.molcel.2005.11.023

[B11] Bowers JL Randell JC Chen S Bell SP ATP hydrolysis by ORC catalyzes reiterative Mcm2-7 assembly at a defined origin of replication Mol Cell 2004 16 6 967 978 15610739 10.1016/j.molcel.2004.11.038

[B12] Evrin C Clarke P Zech J Lurz R Sun J Uhle S Li H Stillman B Speck C A double-hexameric MCM2-7 complex is loaded onto origin DNA during licensing of eukaryotic DNA replication Proc Natl Acad Sci U S A 2009 106 48 20240 20245 19910535 10.1073/pnas.0911500106 PMC2787165

[B13] Remus D Beuron F Tolun G Griffith JD Morris EP Diffley JF Concerted loading of Mcm2-7 double hexamers around DNA during DNA replication origin licensing Cell 2009 139 4 719 730 19896182 10.1016/j.cell.2009.10.015 PMC2804858

[B14] Lei M Kawasaki Y Young MR Kihara M Sugino A Tye BK Mcm2 is a target of regulation by Cdc7-Dbf4 during the initiation of DNA synthesis Genes Dev 1997 11 24 3365 3374 9407029 10.1101/gad.11.24.3365 PMC316824

[B15] Sheu YJ Stillman B Cdc7-Dbf4 phosphorylates MCM proteins via a docking site-mediated mechanism to promote S phase progression Mol Cell 2006 24 1 101 113 17018296 10.1016/j.molcel.2006.07.033 PMC2923825

[B16] Francis LI Randell JC Takara TJ Uchima L Bell SP Incorporation into the prereplicative complex activates the Mcm2-7 helicase for Cdc7-Dbf4 phosphorylation Genes Dev 2009 23 5 643 654 19270162 10.1101/gad.1759609 PMC2658526

[B17] Randell JC Fan A Chan C Francis LI Heller RC Galani K Bell SP Mec1 is one of multiple kinases that prime the Mcm2-7 helicase for phosphorylation by Cdc7 Mol Cell 2010 40 3 353 363 21070963 10.1016/j.molcel.2010.10.017 PMC3021128

[B18] Sheu YJ Stillman B The Dbf4-Cdc7 kinase promotes S phase by alleviating an inhibitory activity in Mcm4 Nature 2010 463 7277 113 117 20054399 10.1038/nature08647 PMC2805463

[B19] Zachariae W Nasmyth K Whose end is destruction: cell division and the anaphase-promoting complex Genes Dev 1999 13 16 2039 2058 10465783 10.1101/gad.13.16.2039

[B20] Cheng L Collyer T Hardy CF Cell cycle regulation of DNA replication initiator factor Dbf4p Mol Cell Biol 1999 19 6 4270 4278 10330168 10.1128/mcb.19.6.4270 PMC104387

[B21] Oshiro G Owens JC Shellman Y Sclafani RA Li JJ Cell cycle control of Cdc7p kinase activity through regulation of Dbf4p stability Mol Cell Biol 1999 19 7 4888 4896 10373538 10.1128/mcb.19.7.4888 PMC84289

[B22] Ferreira MF Santocanale C Drury LS Diffley JF Dbf4p, an essential S phase-promoting factor, is targeted for degradation by the anaphase-promoting complex Mol Cell Biol 2000 20 1 242 248 10594027 10.1128/mcb.20.1.242-248.2000 PMC85080

[B23] Eytan E Moshe Y Braunstein I Hershko A Roles of the anaphase-promoting complex/cyclosome and of its activator Cdc20 in functional substrate binding Proc Natl Acad Sci U S A 2006 103 7 2081 2086 16455800 10.1073/pnas.0510695103 PMC1413726

[B24] Gambus A Jones RC Sanchez-Diaz A Kanemaki M van Deursen F Edmondson RD Labib K GINS maintains association of Cdc45 with MCM in replisome progression complexes at eukaryotic DNA replication forks Nat Cell Biol 2006 8 4 358 366 16531994 10.1038/ncb1382

[B25] Kanemaki M Labib K Distinct roles for Sld3 and GINS during establishment and progression of eukaryotic DNA replication forks EMBO J 2006 25 8 1753 1763 16601689 10.1038/sj.emboj.7601063 PMC1440835

[B26] Zegerman P Diffley JF Phosphorylation of Sld2 and Sld3 by cyclin-dependent kinases promotes DNA replication in budding yeast Nature 2007 445 7125 281 285 17167417 10.1038/nature05432

[B27] Bruck I Kaplan DL Origin Single-stranded DNA Releases Sld3 Protein from the Mcm2-7 Complex, Allowing the GINS Tetramer to Bind the Mcm2-7 Complex J Biol Chem 2011 286 21 18602 18613 21460226 10.1074/jbc.M111.226332 PMC3099676

[B28] Dutta A Bell SP Initiation of DNA replication in eukaryotic cells Annu Rev Cell Dev Biol 1997 13 293 332 9442876 10.1146/annurev.cellbio.13.1.293

[B29] Elsasser S Chi Y Yang P Campbell JL Phosphorylation controls timing of Cdc6p destruction: A biochemical analysis Mol Biol Cell 1999 10 10 3263 3277 10512865 10.1091/mbc.10.10.3263 PMC25589

[B30] Drury LS Perkins G Diffley JF The cyclin-dependent kinase Cdc28p regulates distinct modes of Cdc6p proteolysis during the budding yeast cell cycle Curr Biol 2000 10 5 231 240 10712901 10.1016/s0960-9822(00)00355-9

[B31] Drury LS Perkins G Diffley JF The Cdc4/34/53 pathway targets Cdc6p for proteolysis in budding yeast EMBO J 1997 16 19 5966 5976 9312054 10.1093/emboj/16.19.5966 PMC1170227

[B32] Nguyen VQ Co C Li JJ Cyclin-dependent kinases prevent DNA re-replication through multiple mechanisms Nature 2001 411 6841 1068 1073 11429609 10.1038/35082600

[B33] Vas A Mok W Leatherwood J Control of DNA rereplication via Cdc2 phosphorylation sites in the origin recognition complex Mol Cell Biol 2001 21 17 5767 5777 11486016 10.1128/MCB.21.17.5767-5777.2001 PMC87296

[B34] Wilmes GM Archambault V Austin RJ Jacobson MD Bell SP Cross FR Interaction of the S-phase cyclin Clb5 with an "RXL" docking sequence in the initiator protein Orc6 provides an origin-localized replication control switch Genes Dev 2004 18 9 981 991 15105375 10.1101/gad.1202304 PMC406289

[B35] Chen S Bell SP CDK prevents Mcm2-7 helicase loading by inhibiting Cdt1 interaction with Orc6 Genes Dev 2011 25 4 363 372 21289063 10.1101/gad.2011511 PMC3042159

[B36] Labib K Diffley JF Kearsey SE G1-phase and B-type cyclins exclude the DNA-replication factor Mcm4 from the nucleus Nat Cell Biol 1999 1 7 415 422 10559985 10.1038/15649

[B37] Nguyen VQ Co C Irie K Li JJ Clb/Cdc28 kinases promote nuclear export of the replication initiator proteins Mcm2-7 Curr Biol 2000 10 4 195 205 10704410 10.1016/s0960-9822(00)00337-7

[B38] Liku ME Nguyen VQ Rosales AW Irie K Li JJ CDK phosphorylation of a novel NLS-NES module distributed between two subunits of the Mcm2-7 complex prevents chromosomal rereplication Mol Biol Cell 2005 16 10 5026 5039 16093348 10.1091/mbc.E05-05-0412 PMC1237101

[B39] Kauffman S Wille JJ The mitotic oscillator in Physarum polycephalum J Theor Biol 1975 55 1 47 93 1239646 10.1016/s0022-5193(75)80108-1

[B40] Hyver C Le Guyader H MPF and cyclin: modelling of the cell cycle minimum oscillator Biosystems 1990 24 2 85 90 2147396 10.1016/0303-2647(90)90001-h

[B41] Goldbeter A A minimal cascade model for the mitotic oscillator involving cyclin and cdc2 kinase Proc Natl Acad Sci U S A 1991 88 20 9107 9111 1833774 10.1073/pnas.88.20.9107 PMC52661

[B42] Tyson JJ Modeling the cell division cycle: cdc2 and cyclin interactions Proc Natl Acad Sci U S A 1991 88 16 7328 7332 1831270 10.1073/pnas.88.16.7328 PMC52288

[B43] Norel R Agur Z A model for the adjustment of the mitotic clock by cyclin and MPF levels Science 1991 251 4997 1076 1078 1825521 10.1126/science.1825521

[B44] Li F Long T Lu Y Ouyang Q Tang C The yeast cell-cycle network is robustly designed Proc Natl Acad Sci U S A 2004 101 14 4781 4786 15037758 10.1073/pnas.0305937101 PMC387325

[B45] Chen KC Calzone L Csikasz-Nagy A Cross FR Novak B Tyson JJ Integrative analysis of cell cycle control in budding yeast Mol Biol Cell 2004 15 8 3841 3862 15169868 10.1091/mbc.E03-11-0794 PMC491841

[B46] Chen KC Csikasz-Nagy A Gyorffy B Val J Novak B Tyson JJ Kinetic analysis of a molecular model of the budding yeast cell cycle Mol Biol Cell 2000 11 1 369 391 10637314 10.1091/mbc.11.1.369 PMC14780

[B47] Klipp E Nordlander B Kruger R Gennemark P Hohmann S Integrative model of the response of yeast to osmotic shock Nat Biotechnol 2005 23 8 975 982 16025103 10.1038/nbt1114

[B48] Ingalls BP Duncker BP Kim DR McConkey BJ Systems level modeling of the cell cycle using budding yeast Cancer Inform 2007 3 357 370 19455254 PMC2675848

[B49] Alarcon T Tindall MJ Modelling cell growth and its modulation of the G1/S transition Bull Math Biol 2007 69 1 197 214 17086369 10.1007/s11538-006-9154-0

[B50] Barberis M Klipp E Vanoni M Alberghina L Cell size at S phase initiation: an emergent property of the G1/S network PLoS Comput Biol 2007 3 4 e64 17432928 10.1371/journal.pcbi.0030064 PMC1851985

[B51] Spiesser TW Klipp E Barberis M A model for the spatiotemporal organization of DNA replication in Saccharomyces cerevisiae Mol Genet Genomics 2009 282 1 25 35 19306105 10.1007/s00438-009-0443-9 PMC2695552

[B52] de Moura AP Retkute R Hawkins M Nieduszynski CA Mathematical modelling of whole chromosome replication Nucleic Acids Res 2010 38 17 5623 5633 20457753 10.1093/nar/gkq343 PMC2943597

[B53] Retkute R Nieduszynski CA de Moura A Dynamics of DNA replication in yeast Phys Rev Lett 2011 107 6 068103 21902372 10.1103/PhysRevLett.107.068103 PMC3671325

[B54] Brummer A Salazar C Zinzalla V Alberghina L Hofer T Mathematical modelling of DNA replication reveals a trade-off between coherence of origin activation and robustness against rereplication PLoS Comput Biol 2010 6 5 e1000783 20485558 10.1371/journal.pcbi.1000783 PMC2869307

[B55] Tanaka S Nakato R Katou Y Shirahige K Araki H Origin association of sld3, sld7, and cdc45 proteins is a key step for determination of origin-firing timing Curr Biol 2011 21 24 2055 2063 22169533 10.1016/j.cub.2011.11.038

[B56] Mantiero D Mackenzie A Donaldson A Zegerman P Limiting replication initiation factors execute the temporal programme of origin firing in budding yeast EMBO J 2011 30 23 4805 4814 22081107 10.1038/emboj.2011.404 PMC3243606

[B57] Donovan S Harwood J Drury LS Diffley JF Cdc6p-dependent loading of Mcm proteins onto pre-replicative chromatin in budding yeast Proc Natl Acad Sci U S A 1997 94 11 5611 5616 9159120 10.1073/pnas.94.11.5611 PMC20826

[B58] Lei M Kawasaki Y Tye BK Physical interactions among Mcm proteins and effects of Mcm dosage on DNA replication in Saccharomyces cerevisiae Mol Cell Biol 1996 16 9 5081 5090 8756666 10.1128/mcb.16.9.5081 PMC231509

[B59] Aparicio OM Weinstein DM Bell SP Components and dynamics of DNA replication complexes in S. cerevisiae: redistribution of MCM proteins and Cdc45p during S phase Cell 1997 91 1 59 69 9335335 10.1016/s0092-8674(01)80009-x

[B60] Gibson DG Bell SP Aparicio OM Cell cycle execution point analysis of ORC function and characterization of the checkpoint response to ORC inactivation in Saccharomyces cerevisiae Genes Cells 2006 11 6 557 573 16716188 10.1111/j.1365-2443.2006.00967.x

[B61] Hopwood B Dalton S Cdc45p assembles into a complex with Cdc46p/Mcm5p, is required for minichromosome maintenance, and is essential for chromosomal DNA replication Proc Natl Acad Sci U S A 1996 93 22 12309 12314 8901577 10.1073/pnas.93.22.12309 PMC37987

[B62] Owens JC Detweiler CS Li JJ CDC45 is required in conjunction with CDC7/DBF4 to trigger the initiation of DNA replication Proc Natl Acad Sci U S A 1997 94 23 12521 12526 9356482 10.1073/pnas.94.23.12521 PMC25024

[B63] Liang C Stillman B Persistent initiation of DNA replication and chromatin-bound MCM proteins during the cell cycle in cdc6 mutants Genes Dev 1997 11 24 3375 3386 9407030 10.1101/gad.11.24.3375 PMC316796

[B64] Forsburg SL Eukaryotic MCM proteins: beyond replication initiation Microbiol Mol Biol Rev 2004 68 1 109 131 15007098 10.1128/MMBR.68.1.109-131.2004 PMC362110

[B65] Raghuraman MK Winzeler EA Collingwood D Hunt S Wodicka L Conway A Lockhart DJ Davis RW Brewer BJ Fangman WL Replication dynamics of the yeast genome Science 2001 294 5540 115 121 11588253 10.1126/science.294.5540.115

[B66] Huh WK Falvo JV Gerke LC Carroll AS Howson RW Weissman JS O'Shea EK Global analysis of protein localization in budding yeast Nature 2003 425 6959 686 691 14562095 10.1038/nature02026

[B67] Ghaemmaghami S Huh WK Bower K Howson RW Belle A Dephoure N O'Shea EK Weissman JS Global analysis of protein expression in yeast Nature 2003 425 6959 737 741 14562106 10.1038/nature02046

[B68] Poddar A Stukenberg PT Burke DJ Two complexes of spindle checkpoint proteins containing Cdc20 and Mad2 assemble during mitosis independently of the kinetochore in Saccharomyces cerevisiae Eukaryot Cell 2005 4 5 867 878 15879521 10.1128/EC.4.5.867-878.2005 PMC1140093

[B69] Schreiber A Stengel F Zhang Z Enchev RI Kong EH Morris EP Robinson CV da Fonseca PC Barford D Structural basis for the subunit assembly of the anaphase-promoting complex Nature 2011 470 7333 227 232 21307936 10.1038/nature09756

[B70] Longtine MS McKenzie A Demarini DJ Shah NG Wach A Brachat A Philippsen P Pringle JR Additional modules for versatile and economical PCR-based gene deletion and modification in Saccharomyces cerevisiae Yeast 1998 14 10 953 961 9717241 10.1002/(SICI)1097-0061(199807)14:10<953::AID-YEA293>3.0.CO;2-U

[B71] Pasero P Duncker BP Schwob E Gasser SM A role for the Cdc7 kinase regulatory subunit Dbf4p in the formation of initiation-competent origins of replication Genes Dev 1999 13 16 2159 2176 10465792 10.1101/gad.13.16.2159 PMC316966

[B72] Gadkar KG Gunawan R Doyle FJ Iterative approach to model identification of biological networks BMC Bioinforma 2005 6 155 10.1186/1471-2105-6-155 PMC1189077 15967022

[B73] Jones DR Prasad AA Chan PK Duncker BP The Dbf4 motif C zinc finger promotes DNA replication and mediates resistance to genotoxic stress Cell Cycle 2010 9 10 2018 2026 20436286 10.4161/cc.9.10.11752

[B74] Zou L Mitchell J Stillman B CDC45, a novel yeast gene that functions with the origin recognition complex and Mcm proteins in initiation of DNA replication Mol Cell Biol 1997 17 2 553 563 9001208 10.1128/mcb.17.2.553 PMC231780

[B75] Yu H Regulation of APC-Cdc20 by the spindle checkpoint Curr Opin Cell Biol 2002 14 6 706 714 12473343 10.1016/s0955-0674(02)00382-4

[B76] Goldar A Marsolier-Kergoat MC Hyrien O Universal temporal profile of replication origin activation in eukaryotes PLoS One 2009 4 6 e5899 19521533 10.1371/journal.pone.0005899 PMC2690853

[B77] McCune HJ Danielson LS Alvino GM Collingwood D Delrow JJ Fangman WL Brewer BJ Raghuraman MK The temporal program of chromosome replication: genomewide replication in clb5{Delta} Saccharomyces cerevisiae Genetics 2008 180 4 1833 1847 18832352 10.1534/genetics.108.094359 PMC2600925

[B78] Hyrien O Marheineke K Goldar A Paradoxes of eukaryotic DNA replication: MCM proteins and the random completion problem Bioessays 2003 25 2 116 125 12539237 10.1002/bies.10208

[B79] Green BM Morreale RJ Ozaydin B Derisi JL Li JJ Genome-wide mapping of DNA synthesis in Saccharomyces cerevisiae reveals that mechanisms preventing reinitiation of DNA replication are not redundant Mol Biol Cell 2006 17 5 2401 2414 16481397 10.1091/mbc.E05-11-1043 PMC1446083

[B80] Chassagnole C Jackson RC Hussain N Bashir L Derow C Savin J Fell DA Using a mammalian cell cycle simulation to interpret differential kinase inhibition in anti-tumour pharmaceutical development Biosystems 2006 83 2–3 91 97 16236428 10.1016/j.biosystems.2005.04.007

[B81] Gerard C Goldbeter A From simple to complex patterns of oscillatory behavior in a model for the mammalian cell cycle containing multiple oscillatory circuits Chaos 2010 20 4 045109 21198121 10.1063/1.3527998

[B82] Novak B Tyson JJ A model for restriction point control of the mammalian cell cycle J Theor Biol 2004 230 4 563 579 15363676 10.1016/j.jtbi.2004.04.039

[B83] Qu Z Weiss JN MacLellan WR Regulation of the mammalian cell cycle: a model of the G1-to-S transition Am J Physiol Cell Physiol 2003 284 2 C349 C364 12388094 10.1152/ajpcell.00066.2002

[B84] Swat M Kel A Herzel H Bifurcation analysis of the regulatory modules of the mammalian G1/S transition Bioinformatics 2004 20 10 1506 1511 15231543 10.1093/bioinformatics/bth110

[B85] Lau E Jiang W Is there a pre-RC checkpoint that cancer cells lack? Cell Cycle 2006 5 15 1602 1606 16880740 10.4161/cc.5.15.3124

[B86] Gavin EJ Song B Wang Y Xi Y Ju J Reduction of Orc6 expression sensitizes human colon cancer cells to 5-fluorouracil and cisplatin PLoS One 2008 3 12 e4054 19112505 10.1371/journal.pone.0004054 PMC2603583

[B87] Gonzalez MA Tachibana KE Laskey RA Coleman N Control of DNA replication and its potential clinical exploitation Nat Rev Cancer 2005 5 2 135 141 15660109 10.1038/nrc1548

[B88] Bicknell LS Bongers EM Leitch A Brown S Schoots J Harley ME Aftimos S Al-Aama JY Bober M Brown PA van Bokhoven H Dean J Edrees AY Feingold M Fryer A Hoefsloot LH Kau N Knoers NV Mackenzie J Opitz JM Sarda P Ross A Temple IK Toutain A Wise CA Wright M Jackson AP Mutations in the pre-replication complex cause Meier-Gorlin syndrome Nat Genet 2011 43 4 356 359 21358632 10.1038/ng.775 PMC3068194

[B89] Bicknell LS Walker S Klingseisen A Stiff T Leitch A Kerzendorfer C Martin CA Yeyati P Al Sanna N Bober M Johnson D Wise C Jackson AP O’Driscoll M Jeggo PA Mutations in ORC1, encoding the largest subunit of the origin recognition complex, cause microcephalic primordial dwarfism resembling Meier-Gorlin syndrome Nat Genet 2011 43 4 350 355 21358633 10.1038/ng.776

[B90] Nieduszynski CA Knox Y Donaldson AD Genome-wide identification of replication origins in yeast by comparative genomics Genes Dev 2006 20 14 1874 1879 16847347 10.1101/gad.385306 PMC1522085

[B91] Feng W Collingwood D Boeck ME Fox LA Alvino GM Fangman WL Raghuraman MK Brewer BJ Genomic mapping of single-stranded DNA in hydroxyurea-challenged yeasts identifies origins of replication Nat Cell Biol 2006 8 2 148 155 16429127 10.1038/ncb1358 PMC1414058

[B92] Wyrick JJ Aparicio JG Chen T Barnett JD Jennings EG Young RA Bell SP Aparicio OM Genome-wide distribution of ORC and MCM proteins in S. cerevisiae: high-resolution mapping of replication origins Science 2001 294 5550 2357 2360 11743203 10.1126/science.1066101

[B93] Varrin AE Prasad AA Scholz RP Ramer MD Duncker BP A mutation in Dbf4 motif M impairs interactions with DNA replication factors and confers increased resistance to genotoxic agents Mol Cell Biol 2005 25 17 7494 7504 16107698 10.1128/MCB.25.17.7494-7504.2005 PMC1190303

[B94] Yan H Gibson S Tye BK Mcm2 and Mcm3, two proteins important for ARS activity, are related in structure and function Genes Dev 1991 5 6 944 957 2044961 10.1101/gad.5.6.944

[B95] Yan H Merchant AM Tye BK Cell cycle-regulated nuclear localization of MCM2 and MCM3, which are required for the initiation of DNA synthesis at chromosomal replication origins in yeast Genes Dev 1993 7 11 2149 2160 8224843 10.1101/gad.7.11.2149

[B96] Detweiler CS Li JJ Cdc6p establishes and maintains a state of replication competence during G1 phase J Cell Sci 1997 110 Pt 6 753 763 9099949 10.1242/jcs.110.6.753

[B97] Bousset K Diffley JF The Cdc7 protein kinase is required for origin firing during S phase Genes Dev 1998 12 4 480 490 9472017 10.1101/gad.12.4.480 PMC316531

