# Supplementary material for: A quantitative model of the initiation of DNA replication in Saccharomyces cerevisiae predicts the effects of system perturbations
Source: BMC Syst Biol. 2012 Jun 27;6:78. doi: 10.1186/1752-0509-6-78 (PMC3439281; doi:10.1186/1752-0509-6-78)
Supplement: Additional file 2 — Figure S2. In vivo chromatin fractionation results for Cdc45 as assayed via Western blotting. [file 1752-0509-6-78-S2.pdf]

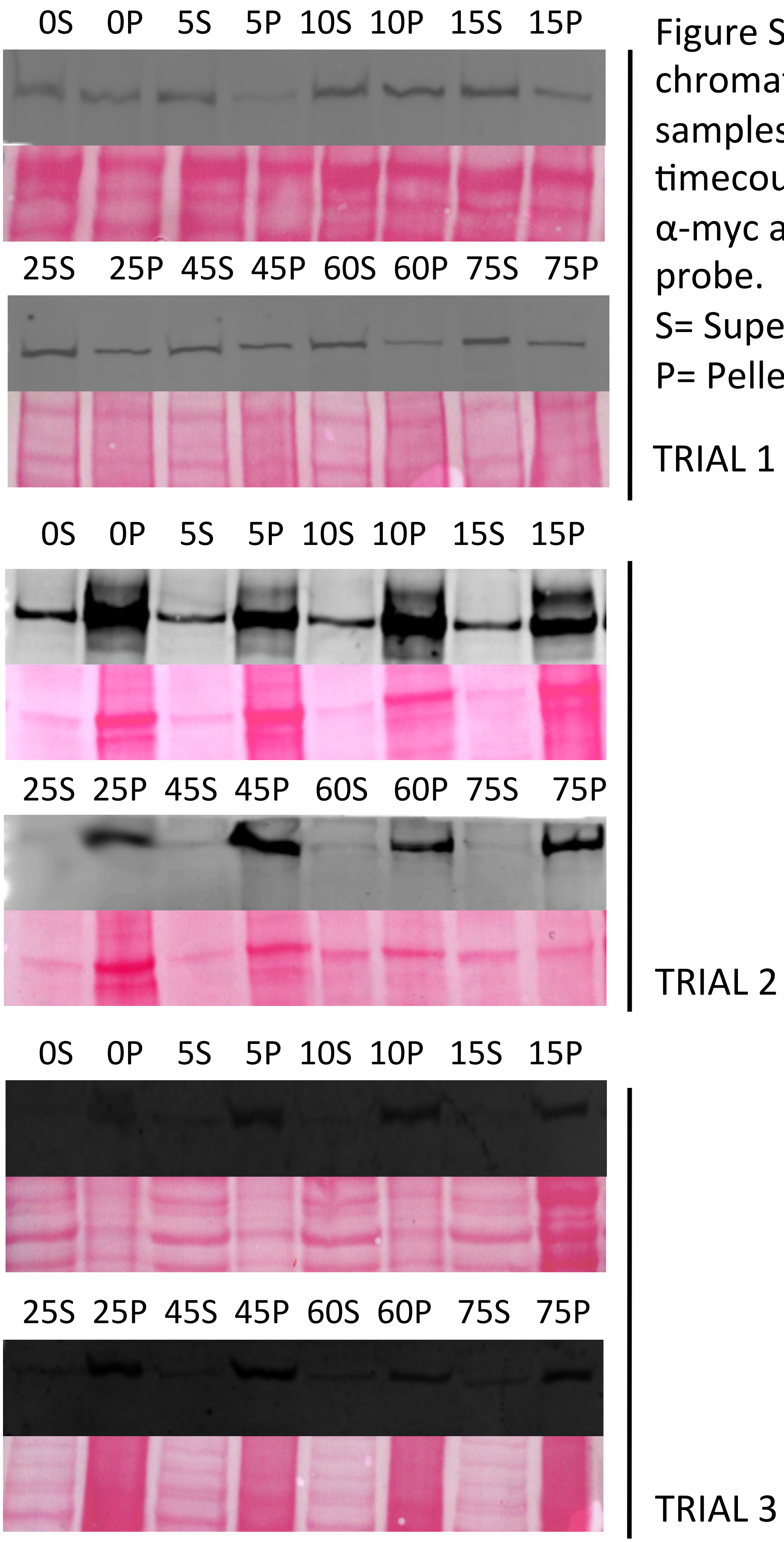

Figure S2. Western blotting of chromatin fractionation samples from three Cd45-myc timecourses. For these blots, α-myc antibody was used to probe.

S= Supernatant (soluble)

P= Pellet (chromatin-bound)
