# Supplementary material for: A quantitative model of the initiation of DNA replication in Saccharomyces cerevisiae predicts the effects of system perturbations
Source: BMC Syst Biol. 2012 Jun 27;6:78. doi: 10.1186/1752-0509-6-78 (PMC3439281; doi:10.1186/1752-0509-6-78)
Supplement: Additional file 3 — Figure S3. In vivo chromatin fractionation results for Mcm2 as assayed via Western blotting. [file 1752-0509-6-78-S3.pdf]

Figure S3. Western blotting of chromatin fractionation samples from three wildtype timecourses. For these blots, α-Mcm2 antibody was used to probe.

S= Supernatant (soluble)

P= Pellet (chromatin-bound)

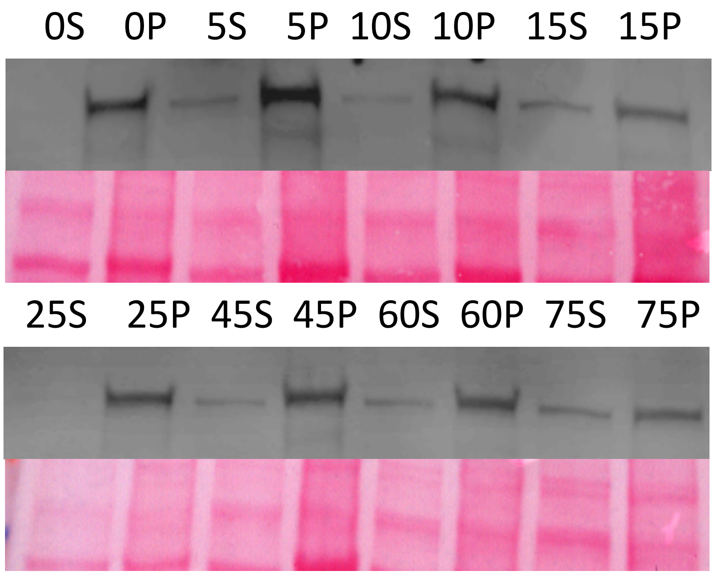

TRIAL 1

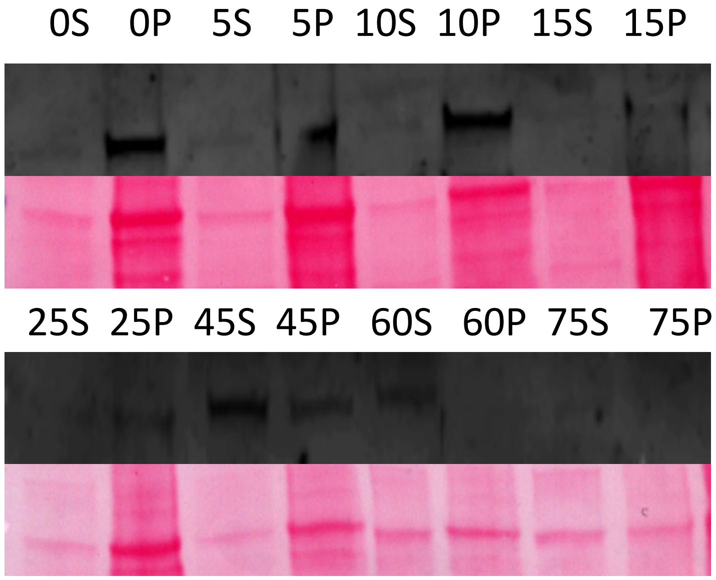

TRIAL 2
