# Supplementary material for: A quantitative model of the initiation of DNA replication in Saccharomyces cerevisiae predicts the effects of system perturbations
Source: BMC Syst Biol. 2012 Jun 27;6:78. doi: 10.1186/1752-0509-6-78 (PMC3439281; doi:10.1186/1752-0509-6-78)
Supplement: Additional file 4 — Table S1.Sample conversion of densitometry values to molecules per cell values for a Cdc45-myc timecourse experiment[66]. [file 1752-0509-6-78-S4.ppt]

## Slide 1
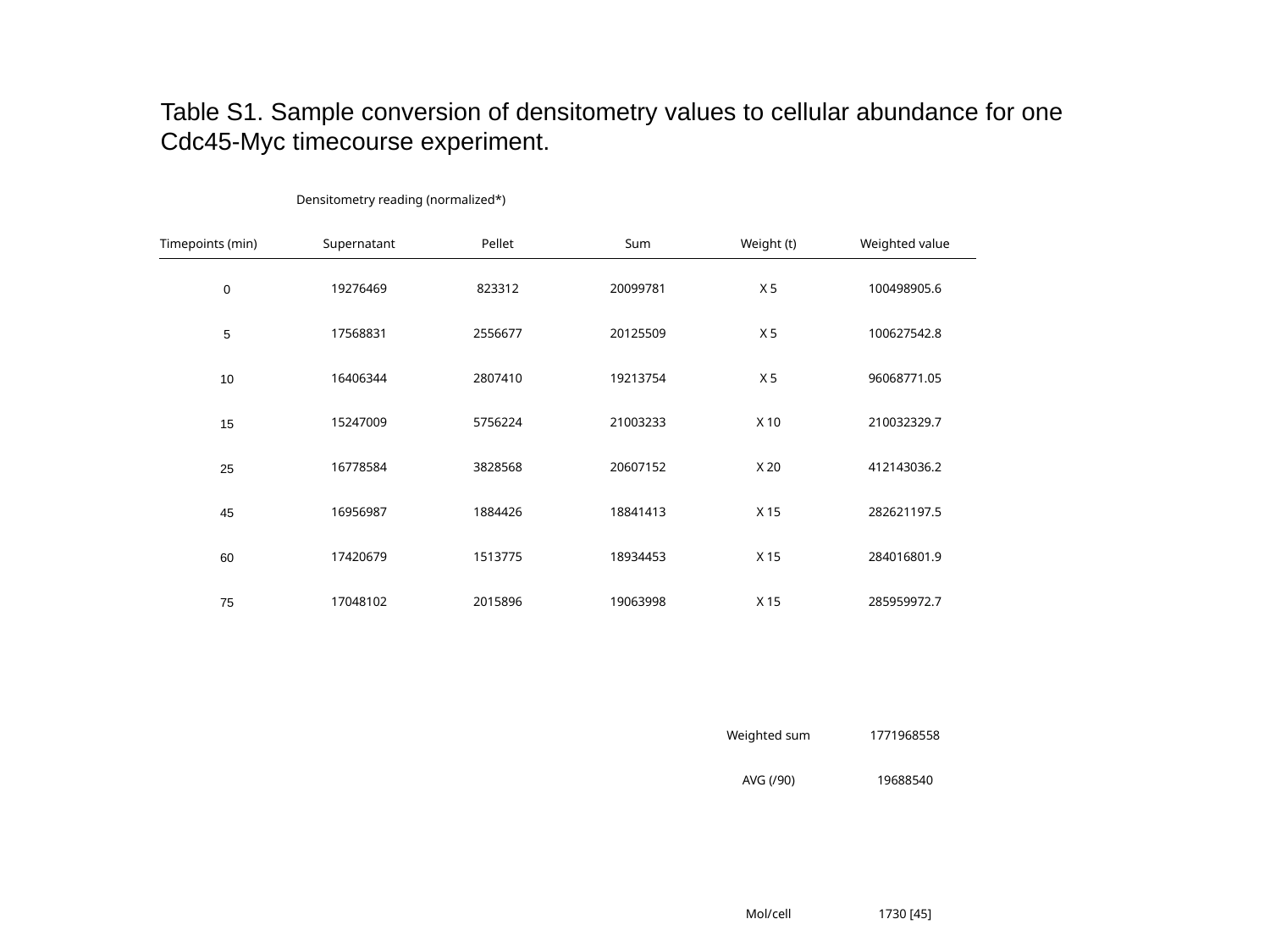

Table S1. Sample conversion of densitometry values to cellular abundance for one Cdc45-Myc timecourse experiment.
| | Densitometry reading (normalized\*) | | | | | |
| --- | --- | --- | --- | --- | --- | --- |
| Timepoints (min) | Supernatant | Pellet | Sum | Weight (t) | Weighted value | |
| 0 | 19276469 | 823312 | 20099781 | X 5 | 100498905.6 | |
| 5 | 17568831 | 2556677 | 20125509 | X 5 | 100627542.8 | |
| 10 | 16406344 | 2807410 | 19213754 | X 5 | 96068771.05 | |
| 15 | 15247009 | 5756224 | 21003233 | X 10 | 210032329.7 | |
| 25 | 16778584 | 3828568 | 20607152 | X 20 | 412143036.2 | |
| 45 | 16956987 | 1884426 | 18841413 | X 15 | 282621197.5 | |
| 60 | 17420679 | 1513775 | 18934453 | X 15 | 284016801.9 | |
| 75 | 17048102 | 2015896 | 19063998 | X 15 | 285959972.7 | |
| | | | | | | |
| | | | | | | |
| | | | | Weighted sum | 1771968558 | |
| | | | | AVG (/90) | 19688540 | |
| | | | | | | |
| | | | | | | |
| \* Values are normalized to intensity of Ponceau S staining as well as concentration ratios of supernatant to pellet | | | | Mol/cell | 1730 [45] | |
| | | | | Scaling factor = | (mol/cell)/AVG = | 8.79E-05 |
| | | | | | | |
| | | | | | | |
| | | | | Multiply each timepoint by scaling factor to generate scaled data: | | |
| | | | | | | |
| | | | | | Supernatant | Pellet |
| | | | | 0 | 1694 | 72 |
| | | | | 5 | 1544 | 225 |
| | | | | 10 | 1442 | 247 |
| | | | | 15 | 1340 | 506 |
| | | | | 25 | 1474 | 336 |
| | | | | 45 | 1490 | 166 |
| | | | | 60 | 1531 | 133 |
| | | | | 75 | 1498 | 177 |
