# Supplementary material for: A quantitative model of the initiation of DNA replication in Saccharomyces cerevisiae predicts the effects of system perturbations
Source: BMC Syst Biol. 2012 Jun 27;6:78. doi: 10.1186/1752-0509-6-78 (PMC3439281; doi:10.1186/1752-0509-6-78)
Supplement: Additional file 5 — Figure S4. Levels of model components when Cdc6, Cdt1 or Dbf4 have been reduced to 10% of their wild-type levels. [file 1752-0509-6-78-S5.pdf]

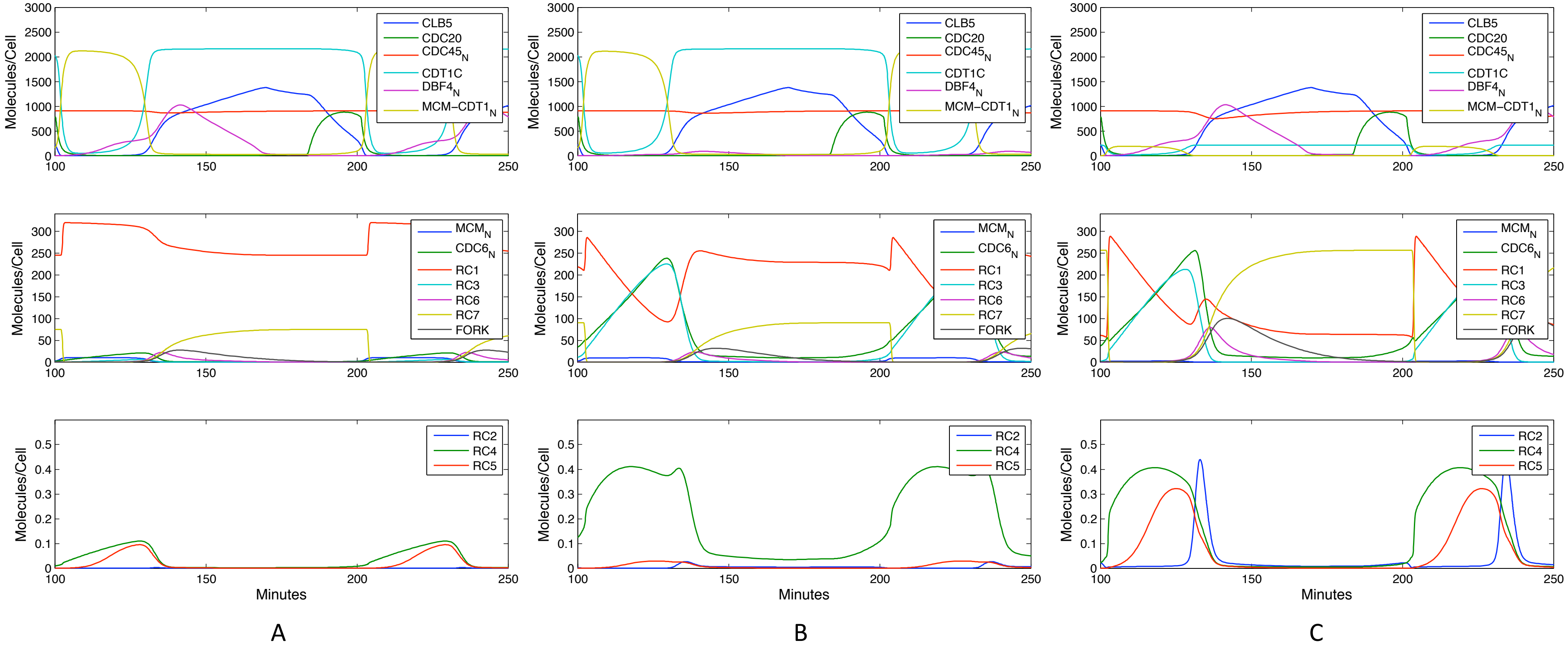


Figure S4. Replication model component levels when Cdc6 (A), Db4f (B) and Cdt1 (C) are reduced to 10% of their wild-type levels.
