# Supplementary material for: A quantitative model of the initiation of DNA replication in Saccharomyces cerevisiae predicts the effects of system perturbations
Source: BMC Syst Biol. 2012 Jun 27;6:78. doi: 10.1186/1752-0509-6-78 (PMC3439281; doi:10.1186/1752-0509-6-78)
Supplement: Additional file 6 — Figure S5. The combination of DNA replication and whole cell cycle models does not alter either’s behaviour in isolation[45]. [file 1752-0509-6-78-S6.ppt]

## Slide 1
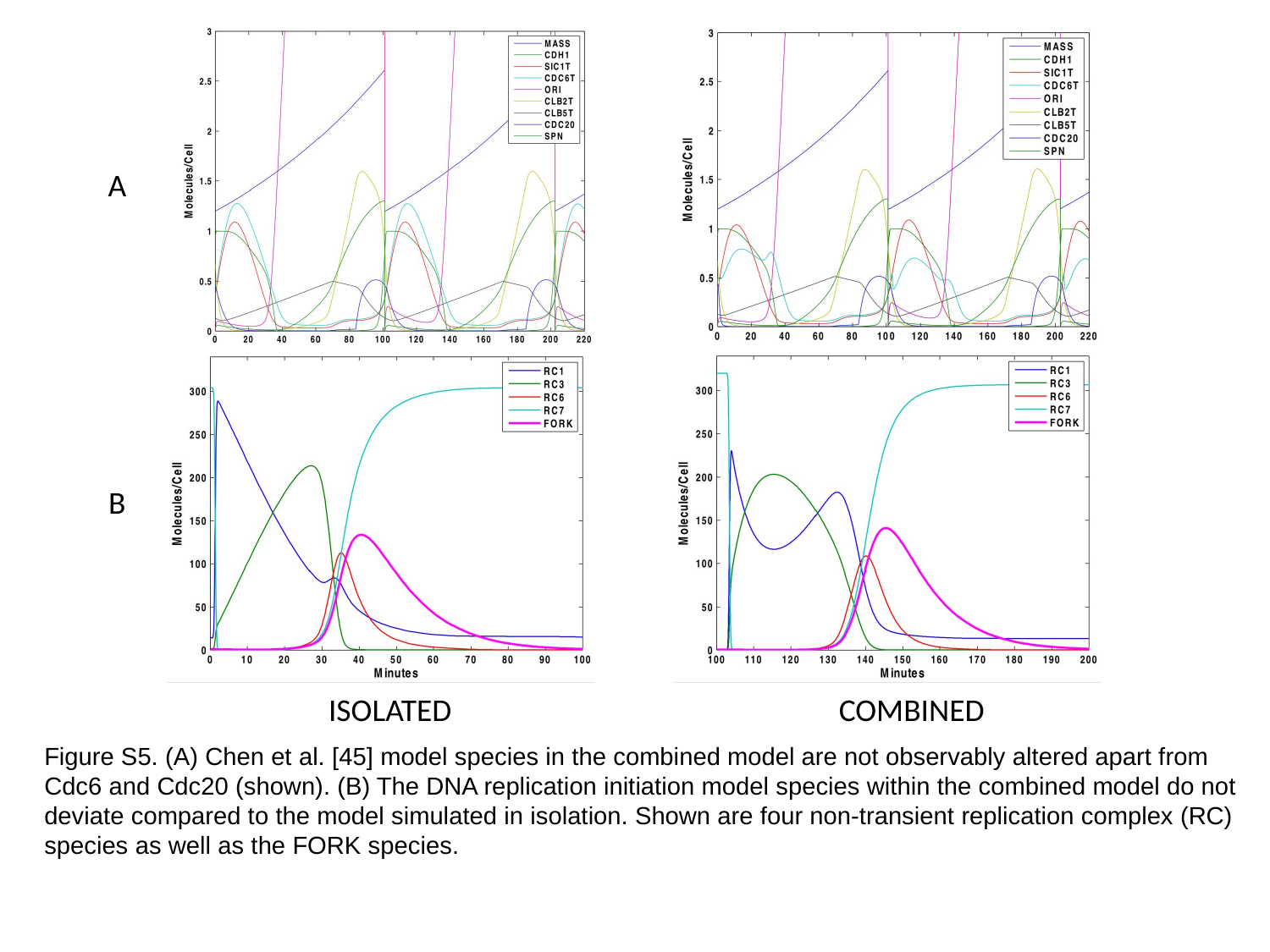

A
B
ISOLATED
COMBINED
Figure S5. (A) Chen et al. [45] model species in the combined model are not observably altered apart from Cdc6 and Cdc20 (shown). (B) The DNA replication initiation model species within the combined model do not deviate compared to the model simulated in isolation. Shown are four non-transient replication complex (RC) species as well as the FORK species.
