# Supplementary material for: A quantitative model of the initiation of DNA replication in Saccharomyces cerevisiae predicts the effects of system perturbations
Source: BMC Syst Biol. 2012 Jun 27;6:78. doi: 10.1186/1752-0509-6-78 (PMC3439281; doi:10.1186/1752-0509-6-78)
Supplement: Additional file 7 — Figure S6.Mutant phenotypes reported in whole cell cycle model are unaltered in the combined model[45]. [file 1752-0509-6-78-S7.ppt]

## Slide 1
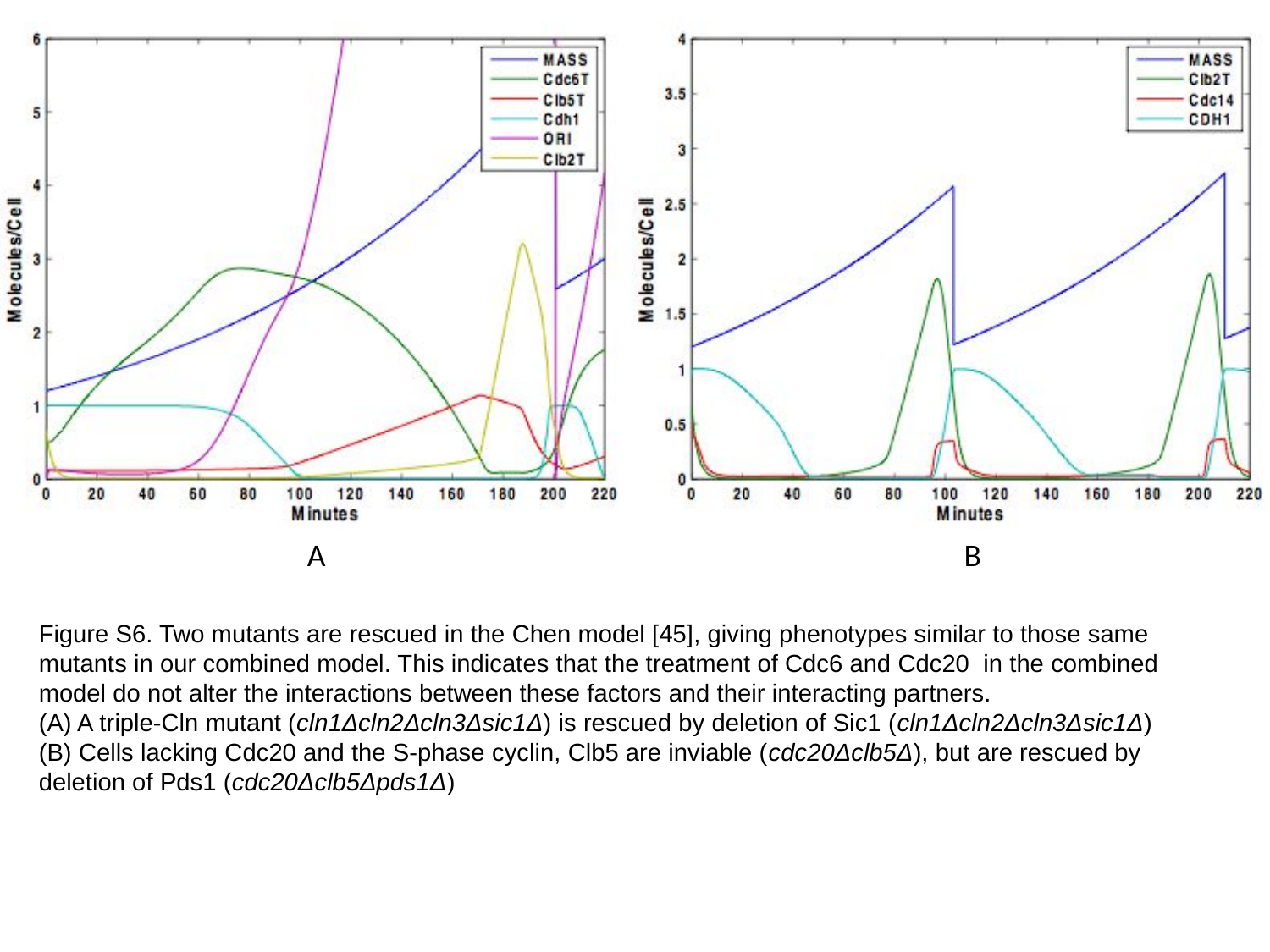

A
B
Figure S6. Two mutants are rescued in the Chen model [45], giving phenotypes similar to those same mutants in our combined model. This indicates that the treatment of Cdc6 and Cdc20 in the combined model do not alter the interactions between these factors and their interacting partners.
(A) A triple-Cln mutant (cln1Δcln2Δcln3Δsic1Δ) is rescued by deletion of Sic1 (cln1Δcln2Δcln3Δsic1Δ)
(B) Cells lacking Cdc20 and the S-phase cyclin, Clb5 are inviable (cdc20Δclb5Δ), but are rescued by deletion of Pds1 (cdc20Δclb5Δpds1Δ)
