# Supplementary material for: A quantitative model of the initiation of DNA replication in Saccharomyces cerevisiae predicts the effects of system perturbations
Source: BMC Syst Biol. 2012 Jun 27;6:78. doi: 10.1186/1752-0509-6-78 (PMC3439281; doi:10.1186/1752-0509-6-78)
Supplement: Additional file 8 — Figure S7. Comparison of Cdc20 time-varying profiles as originally modeled by Chen [24] versus our modified version [45]. [file 1752-0509-6-78-S8.ppt]

## Slide 1
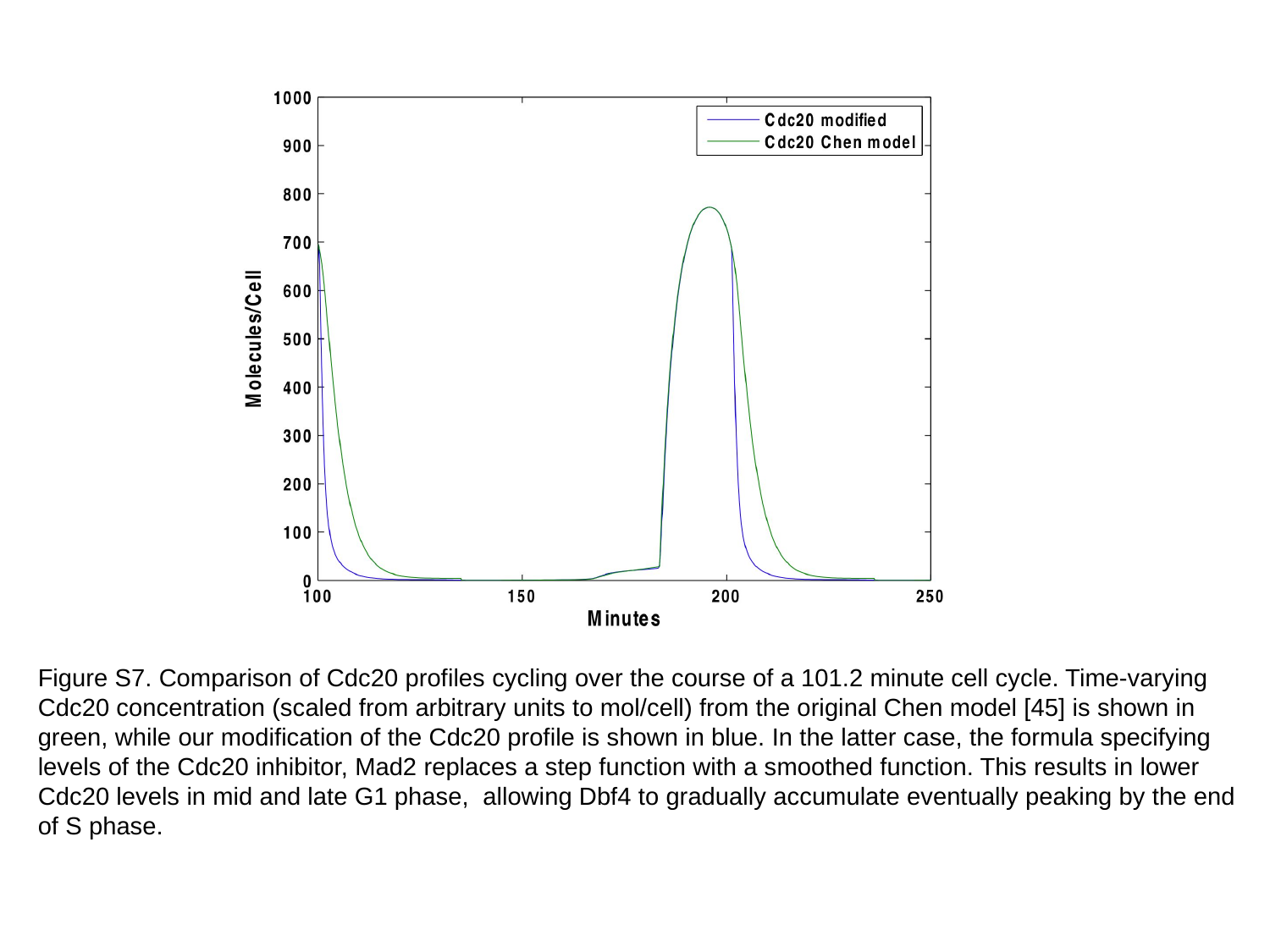

Figure S7. Comparison of Cdc20 profiles cycling over the course of a 101.2 minute cell cycle. Time-varying Cdc20 concentration (scaled from arbitrary units to mol/cell) from the original Chen model [45] is shown in green, while our modification of the Cdc20 profile is shown in blue. In the latter case, the formula specifying levels of the Cdc20 inhibitor, Mad2 replaces a step function with a smoothed function. This results in lower Cdc20 levels in mid and late G1 phase, allowing Dbf4 to gradually accumulate eventually peaking by the end of S phase.
