# Supplementary material for: A quantitative model of the initiation of DNA replication in Saccharomyces cerevisiae predicts the effects of system perturbations
Source: BMC Syst Biol. 2012 Jun 27;6:78. doi: 10.1186/1752-0509-6-78 (PMC3439281; doi:10.1186/1752-0509-6-78)
Supplement: Additional file 9 — Table S2. Summary of simulated cell cycle mutants in the internal DNA replication initiation model. Table S3. Comparison of cell cycle mutants simulated by Chen et al. [45] and by the combined model [7,45,55,60,73,74,94-97]. [file 1752-0509-6-78-S9.ppt]

## Slide 1
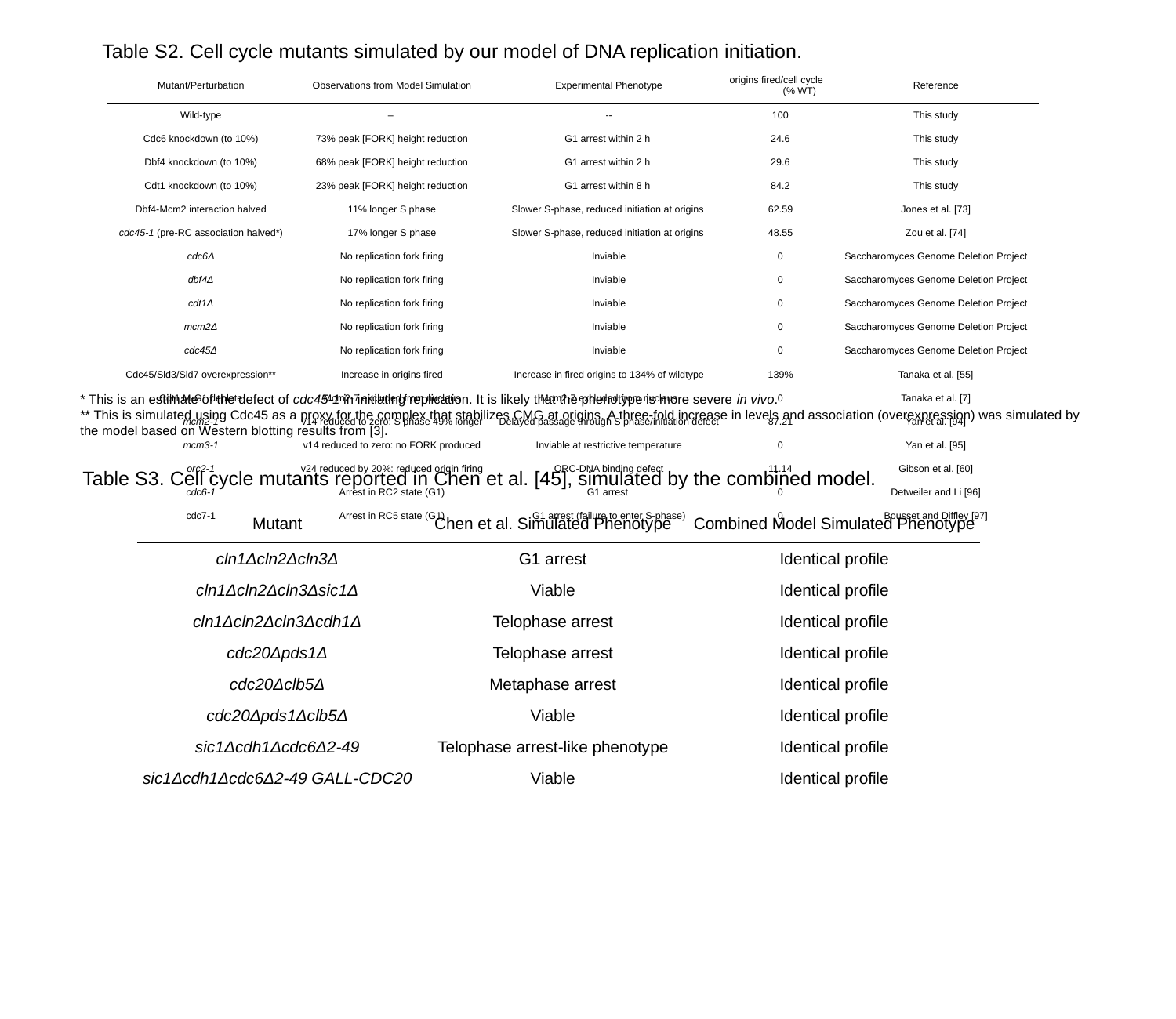

Table S2. Cell cycle mutants simulated by our model of DNA replication initiation.
| Mutant/Perturbation | Observations from Model Simulation | Experimental Phenotype | origins fired/cell cycle (% WT) | Reference |
| --- | --- | --- | --- | --- |
| Wild-type | – | -- | 100 | This study |
| Cdc6 knockdown (to 10%) | 73% peak [FORK] height reduction | G1 arrest within 2 h | 24.6 | This study |
| Dbf4 knockdown (to 10%) | 68% peak [FORK] height reduction | G1 arrest within 2 h | 29.6 | This study |
| Cdt1 knockdown (to 10%) | 23% peak [FORK] height reduction | G1 arrest within 8 h | 84.2 | This study |
| Dbf4-Mcm2 interaction halved | 11% longer S phase | Slower S-phase, reduced initiation at origins | 62.59 | Jones et al. [73] |
| cdc45-1 (pre-RC association halved\*) | 17% longer S phase | Slower S-phase, reduced initiation at origins | 48.55 | Zou et al. [74] |
| cdc6Δ | No replication fork firing | Inviable | 0 | Saccharomyces Genome Deletion Project |
| dbf4Δ | No replication fork firing | Inviable | 0 | Saccharomyces Genome Deletion Project |
| cdt1Δ | No replication fork firing | Inviable | 0 | Saccharomyces Genome Deletion Project |
| mcm2Δ | No replication fork firing | Inviable | 0 | Saccharomyces Genome Deletion Project |
| cdc45Δ | No replication fork firing | Inviable | 0 | Saccharomyces Genome Deletion Project |
| Cdc45/Sld3/Sld7 overexpression\*\* | Increase in origins fired | Increase in fired origins to 134% of wildtype | 139% | Tanaka et al. [55] |
| Cdt1 M-G1 deplete | Mcm2-7 excluded from nucleus | Mcm2-7 excluded from nucleus | 0 | Tanaka et al. [7] |
| mcm2-1 | v14 reduced to zero: S phase 49% longer | Delayed passage through S phase/initiation defect | 87.21 | Yan et al. [94] |
| mcm3-1 | v14 reduced to zero: no FORK produced | Inviable at restrictive temperature | 0 | Yan et al. [95] |
| orc2-1 | v24 reduced by 20%: reduced origin firing | ORC-DNA binding defect | 11.14 | Gibson et al. [60] |
| cdc6-1 | Arrest in RC2 state (G1) | G1 arrest | 0 | Detweiler and Li [96] |
| cdc7-1 | Arrest in RC5 state (G1) | G1 arrest (failure to enter S-phase) | 0 | Bousset and Diffley [97] |
* This is an estimate of the defect of cdc45-1 in initiating replication. It is likely that the phenotype is more severe in vivo.
** This is simulated using Cdc45 as a proxy for the complex that stabilizes CMG at origins. A three-fold increase in levels and association (overexpression) was simulated by the model based on Western blotting results from [3].
Table S3. Cell cycle mutants reported in Chen et al. [45], simulated by the combined model.
| Mutant | Chen et al. Simulated Phenotype | Combined Model Simulated Phenotype |
| --- | --- | --- |
| cln1Δcln2Δcln3Δ | G1 arrest | Identical profile |
| cln1Δcln2Δcln3Δsic1Δ | Viable | Identical profile |
| cln1Δcln2Δcln3Δcdh1Δ | Telophase arrest | Identical profile |
| cdc20Δpds1Δ | Telophase arrest | Identical profile |
| cdc20Δclb5Δ | Metaphase arrest | Identical profile |
| cdc20Δpds1Δclb5Δ | Viable | Identical profile |
| sic1Δcdh1Δcdc6Δ2-49 | Telophase arrest-like phenotype | Identical profile |
| sic1Δcdh1Δcdc6Δ2-49 GALL-CDC20 | Viable | Identical profile |
